# Engineered extracellular vesicle-based sonotheranostics for dual stimuli-sensitive drug release and photoacoustic imaging-guided chemo-sonodynamic cancer therapy

**DOI:** 10.7150/thno.65516

**Published:** 2022-01-01

**Authors:** Thuy Giang Nguyen Cao, Ji Hee Kang, Wangyu Kim, Junha Lim, Su Jin Kang, Jae Young You, Quan Truong Hoang, Won Jong Kim, Won Jong Rhee, Chulhong Kim, Young Tag Ko, Min Suk Shim

**Affiliations:** 1 Division of Bioengineering, Incheon National University, Incheon 22012, Republic of Korea.; 2 College of Pharmacy, Gachon University, Incheon 21936, Republic of Korea.; 3 Department of Electrical Engineering, Convergence IT Engineering, and Mechanical Engineering, Medical Device Innovation Center, Pohang University of Science and Technology (POSTECH), Pohang 37673, Republic of Korea.; 4 Department of Chemistry, Pohang University of Science and Technology (POSTECH), Pohang 37673, Republic of Korea.; 5 Research Center for Bio Materials & Process Development, Incheon National University, 119 Academy-ro, Yeonsu-gu, Incheon 22012, Republic of Korea.

**Keywords:** extracellular vesicle, sonodynamic cancer therapy, sonotheranostics, pH-sensitive release, photoacoustic imaging

## Abstract

Sonodynamic therapy has shown promise as an effective alternative to conventional photodynamic therapy owing to its ability to treat deep-seated tumors. However, the development of stimuli-responsive sonosensitizers with high biocompatibility faces a significant challenge.

**Methods:** In this study, we developed dual stimuli-responsive sonosensitizers with desirable biosafety using extracellular vesicles (EVs), a class of naturally occurring nanoparticles. Indocyanine green (ICG), which functions as both a sonosensitizer and photoacoustic (PA) imaging agent, was loaded into EVs, together with paclitaxel (PTX) and sodium bicarbonate (SBC), to achieve pH-responsive PA imaging-guided chemo-sonodynamic combination therapy.

**Results:** The EVs significantly improved the cellular uptake of ICG, thus triggering enhanced sonodynamic effects in breast cancer cells. SBC-, ICG-, and PTX-loaded EV [SBC-EV(ICG/PTX)] efficiently released the PTX in response to acidic pH in the endo/lysosomes because CO_2_ bubbles generated from the SBC caused the EV membranes to burst. The drug release was further facilitated by ultrasound (US) treatment, demonstrating dual pH/US-responsive drug release. The ICG- and PTX-loaded EVs exhibited efficient anticancer activity against breast tumor cells owing to the combination of chemo-sonodynamic therapy. High-resolution PA imaging visualized the preferential tumor accumulation of SBC-EV(ICG/PTX) in tumor-bearing mice. Notably, a single intravenous injection of SBC-EV(ICG/PTX) with US irradiation significantly suppressed tumor growth in mice without systemic toxicity.

**Conclusions:** Our findings demonstrate that dual stimuli-responsive SBC-EV(ICG/PTX) are promising sonotheranostic nanoplatforms for safe and efficient chemo-sonodynamic combination cancer therapy and photoacoustic imaging.

## Introduction

Oxidative stress-inducing anticancer therapies by increasing the concentration of reactive oxygen species (ROS) have recently emerged as an attractive option for efficient cancer treatment 1-3. Based on this approach, photodynamic therapy (PDT), in which the activation of photosensitizers with light generates cytotoxic ROS via energy transfer to neighboring oxygen molecules, has garnered significant attention for targeted and effective cancer therapy 4. Although PDT has shown selective tumor eradication and low systemic toxicity, its clinical application has been challenged by its limited light penetration in tissues 5. Sonodynamic therapy (SDT), which adopts sonosensitizers to generate ROS under ultrasound (US), enables the treatment of various kinds of deep-seated tumors owing to the deep tissue penetration of US. Therefore, SDT has emerged as a promising alternative 6, 7.

Although the outcome of SDT is greatly affected by the potency of sonosensitizers, the development of effective and safe sonosensitizers, considering the quantum yields of ROS production, biocompatibility, cancer-selectivity, and long-term stability, remains a significant challenge 8. The emergence and rapid development of nanomaterials together with sonosensitizers has gained considerable attention because they can significantly improve the physicochemical properties and anticancer activities of sonosensitizers by addressing their critical limitations 9, 10. It has been reported that nanocarriers, including micelles, liposomes, and polymeric nanoparticles, can enhance biocompatibility, intracellular delivery, cancer-specificity, and *in vivo* pharmacokinetic properties of sonosensitizers 9-11.

Recently, several studies have demonstrated that combination cancer therapy can realize synergistic effects in cancer treatment and reduce the therapeutic doses of anticancer drugs, thereby resulting in minimal side effects 12-15. Combination of chemotherapy with SDT has shown great promise to achieve synergistic therapeutic outcomes for cancer treatment 16. SDT has also been demonstrated to overcome drug resistance of chemotherapy by improving the cellular internalization of drugs and reducing the expression levels of ATP-binding cassette transporters such as P-glycoprotein 16. To obtain effective chemo-sonodynamic combined therapeutic efficacy, the use of nanocarriers that can simultaneously and preferentially deliver both sonosensitizers and anticancer drugs into cancer cells is desirable 16. In addition, engineering nanocarriers that can facilitate the release of anticancer drugs in response to specific stimuli in tumors is crucial to maximize anticancer efficacy while avoiding unwanted drug release in surrounding normal tissues. Despite the enormous potential of stimuli-responsive nanocarriers for chemo-sonodynamic combination therapy, their inherent toxicity and immunogenicity have limited their widespread use in clinical practice 9. Therefore, to facilitate the translation of chemo-sonodynamic combination therapy in clinical practice, the development of highly biocompatible stimuli-responsive nanocarriers is crucial.

In recent years, extracellular vesicles (EVs), nanoscale cell-derived membrane vesicles, have emerged as safe and versatile nanoplatforms for drug delivery 17, 18. These endogenous nanoplatforms ensure exceptional biocompatibility, prolonged blood circulation, and minimal or no immunogenicity 19, 20. In addition, nanoscale EVs can preferentially accumulate into tumors owing to enhanced permeability and retention (EPR) effects 21. Thus, EVs could be effective nanoplatforms for the selective delivery of sonosensitizers and anticancer drugs into tumor legions, ensuring a synergistic and tumor-specific combination anticancer therapy.

Optical imaging for the early diagnosis of diseases and treatment evaluation is crucial for personalized medicine 22, 23. Theranostic nanocarriers that integrate therapy and diagnostics in a single platform have exhibited the substantial potential to play pivotal roles in personalized cancer therapy, including early diagnosis, personalized treatment, and treatment monitoring 24, 25. Photoacoustic (PA) imaging, which obtains images via conversion of photon energy into acoustic pressure waves, has rapidly emerged as an attractive imaging modality for clinical cancer diagnosis 26-28 owing to the imaging of deep tissues at high spatial resolution 29-32. Therefore, the development of a theranostic agent that can achieve PA imaging-guided cancer therapy is crucial because it can enhance the efficacy and safety of cancer treatment simultaneously.

In this study, we developed sonosensitizer and anticancer drug-loaded EVs to achieve simultaneous PA imaging and combination chemo-SDT. A near-infrared (NIR)-absorbing indocyanine green (ICG), a biocompatible theranostic dye approved by the Food and Drug Administration (FDA) 33, 34, was loaded into the EVs to function as both a sonosensitizer and PA imaging agent. The anticancer drug, paclitaxel (PTX), was also encapsulated into the EVs to realize combined chemo-SDT. In addition, to render pH-sensitive EVs, NaHCO_3_ (sodium bicarbonate, SBC) was incorporated into the EVs together with ICG and PTX. In acidic environments, the entrapped SBC in the EVs decomposes spontaneously, rapidly generating CO_2_ bubbles 35, which can cause EV membranes to burst and readily release the payloads. Such pH-responsive EVs could be effective for the intracellular release of drugs once they enter the acidic endo/lysosomes after endocytosis into cancer cells.

These pH-sensitive EVs encapsulating ICG and PTX exhibited several salient features. The encapsulation of ICG into EVs can address the intrinsic drawbacks of ICG, such as low aqueous stability, rapid systemic clearance, and lack of cancer-targeted delivery 36, 37. Notably, EV-based drug delivery systems have better safety profiles than other synthetic nanocarriers because they solely comprise biocompatible materials [that is, EVs and cargo (i.e., ICG)]. These pH-sensitive EVs can facilitate the endo/lysosomal escape and cytoplasmic release of drugs via the acid-destabilization of the EVs in response to the acidic pH in the endo/lysosomal compartments, thereby enabling enhanced anticancer effects. Finally, nanoscale ICG-loaded EVs can function as cancer-targeted theranostic nanocarriers to achieve PA imaging-guided SDT against cancer.

**Figure 1:**
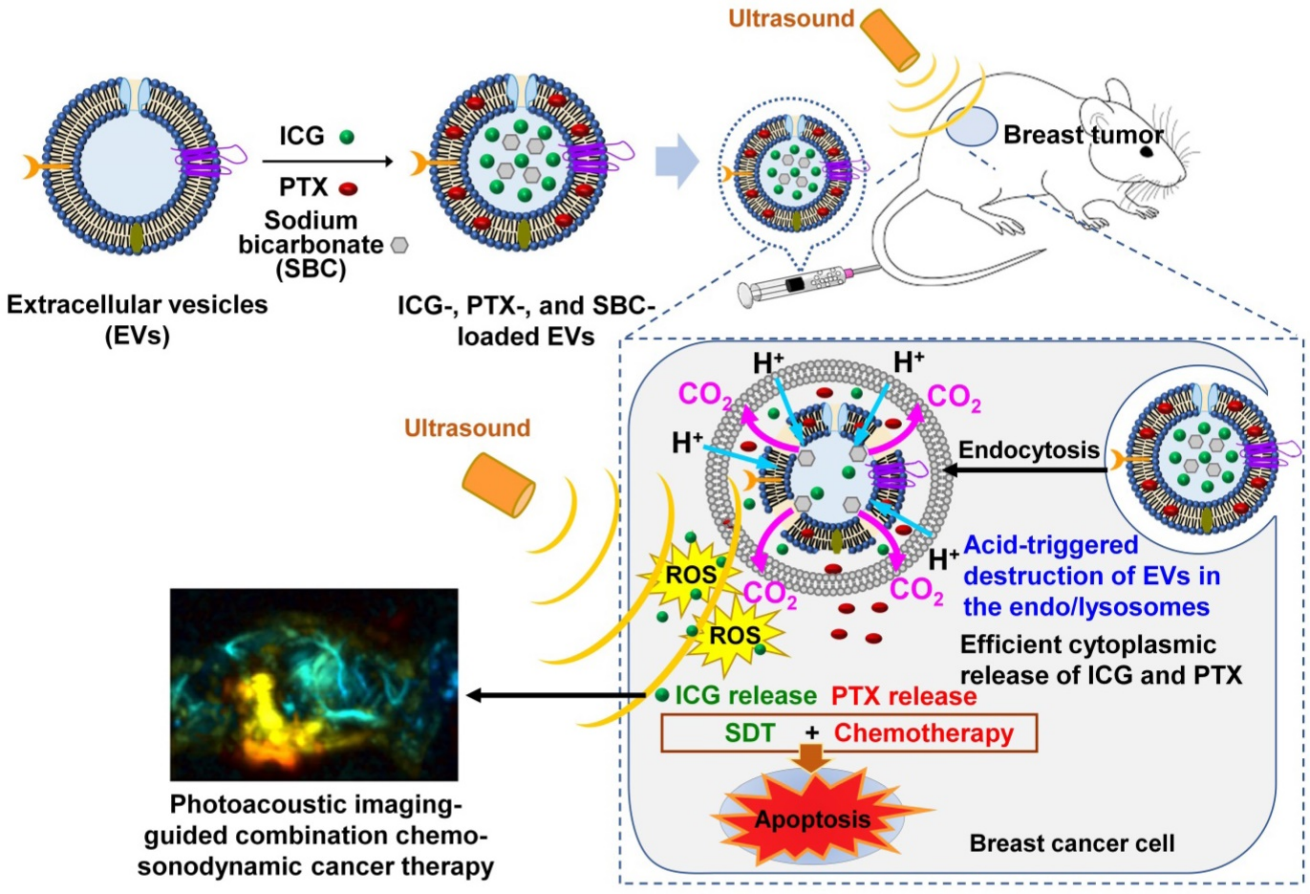
** Schematic illustration of the sonotheranostic action of dual pH/US-responsive EVs co-encapsulating ICG, PTX, and SBC.** Once the EVs enter the cancer cells via endocytosis, the acidic pH in the endo/lysosomes facilitates the release of ICG and PTX owing to bursting effects triggered by the generation of CO_2_ bubbles from the SBC. After the cytoplasmic release of ICG and PTX, US irradiation triggers sonodynamic effects by generating intracellular ROS, subsequently triggering the apoptosis of breast cancer cells. The ICG-loaded EVs generate PA imaging signals, thus enabling PA imaging-guided chemo-sonodynamic combination cancer therapy.

Figure 1 presents a schematic of the PA imaging-guided combination chemo-SDT using pH-sensitive SBC-loaded EVs comprising ICG and PTX [SBC-EV(ICG/PTX)]. Once the EVs enter the cancer cells via endocytosis, the acidic pH in the endo/lysosomes facilitates the release of ICG and PTX by the generation of CO_2_ bubbles from the SBC. After the cytoplasmic release of ICG and PTX, US irradiation activates the ICG to generate ROS in breast cancer cells, which subsequently triggers apoptosis. PTX also induces apoptosis, resulting in synergistic anticancer effects via a combination of chemotherapy and SDT. The *in vivo* biodistribution of pH-sensitive EVs can be monitored by PA imaging. To the best of our knowledge, this is the first study to utilize dual pH/US-sensitive EVs for PA imaging-guided, cancer-targeted combination chemo-SDT. Dual stimuli-sensitive EVs have great potential for clinical applications owing to their high biocompatibility and anticancer efficacies.

## Results and Discussion

### Design, preparation, and characterization of drug-loaded EVs

A significant limitation to anticancer drug delivery is the selective delivery of therapeutic drugs to tumor areas 38. It is well known that nanoscale drug carriers can selectively accumulate into tumor lesions via EPR effects 38-40. However, artificial nanocarriers face certain critical challenges, such as systemic toxicity and nonspecific uptake by the reticuloendothelial system 41. In contrast to synthetic nanocarriers, EVs are naturally occurring nanoparticles with excellent biocompatibility and long-term blood circulatory capability 42. In this study, EVs were engineered as sonotheranostic nanocarriers for safe and efficient delivery of sonosensitizers (i.e., ICG) and chemo drugs (i.e., PTX) into tumors.

To prepare ICG- and PTX-loaded EVs, EVs were obtained from human embryonic kidney HEK-293T cells. It was reported that EVs obtained from HEK-293T cells are benign in terms of *in vivo* immunogenicity and toxicity 43. HEK-293T cell-derived EVs are known to contain low levels of proteins and RNAs associated with the disease- or cancer-related pathways 44. HEK-293T cell-derived EVs in phosphate-buffered saline (PBS) were solely mixed with an ICG solution or together with a PTX solution in dimethyl sulfoxide (DMSO). DMSO was used as a permeability enhancer by increasing the solubility of drugs and their permeability across the lipid membrane of EVs 45, 46. It has been reported that DMSO can induce water pores in lipid bilayers and thus enhance the membrane permeability of both hydrophilic and hydrophobic molecules 46. The loading capacities of ICG and PTX into EVs were proportional to the concentrations of DMSO (Table 1). 4% (v/v) DMSO in PBS was used for drug encapsulation because higher DMSO concentrations significantly destroyed EVs.

**Figure 2:**
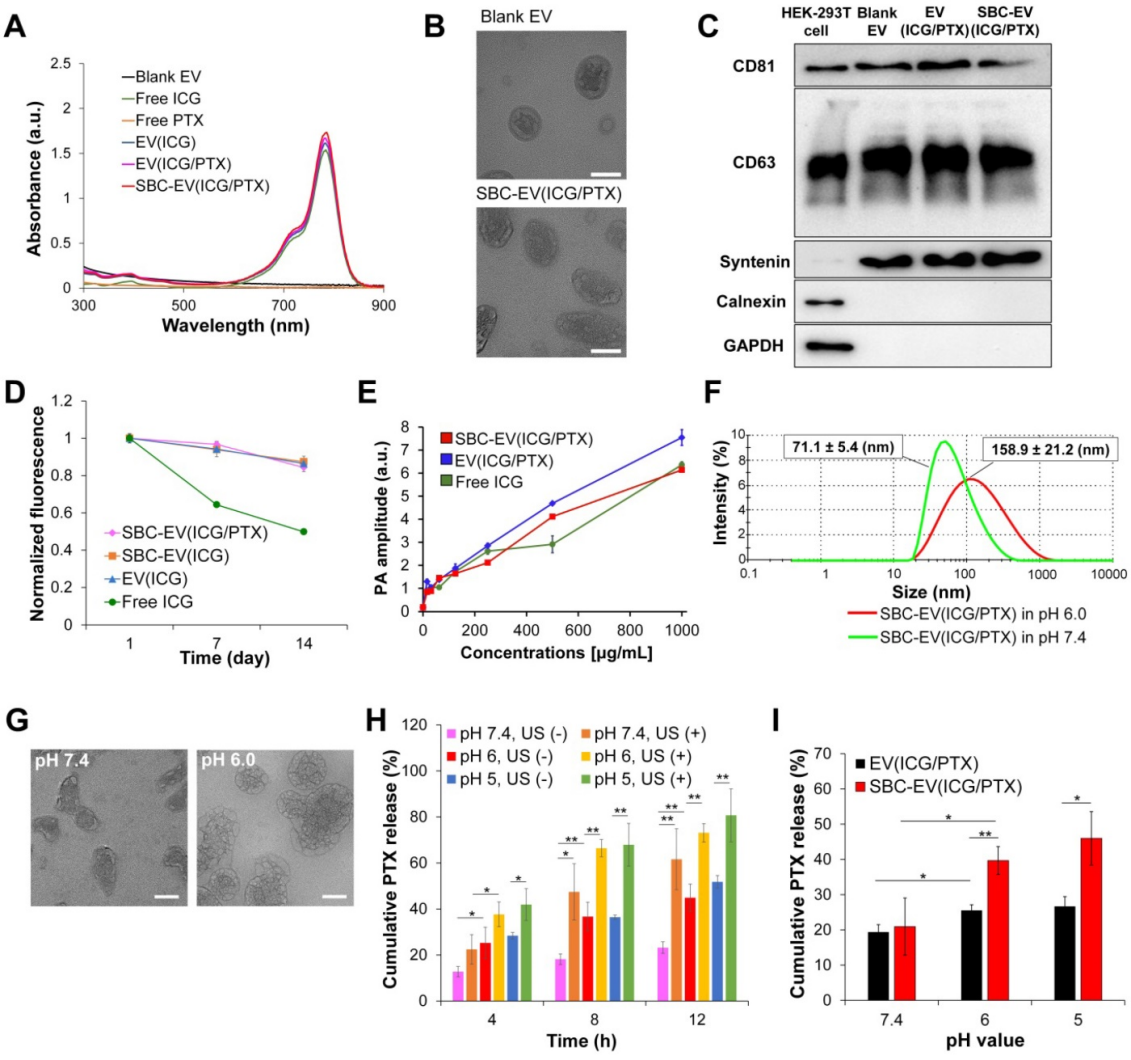
** (A)** Absorption spectra of blank EV, free ICG, free PTX, EV(ICG), EV(ICG/PTX), and SBC-EV(ICG/PTX) samples.** (B)** TEM images of blank EV and SBC-EV(ICG/PTX). Scale bars indicate 50 nm. **(C)** Western blot of typical exosomal markers (CD81, CD63, and syntenin) from HEK-293T cells and various EV samples. Calnexin and GAPDH, housekeeping genes, were used as a control. **(D)** Changes in fluorescence intensity of free ICG, EV(ICG), SBC-EV(ICG), and SBC-EV(ICG/PTX) after incubation in PBS (4 °C) for 14 days. **(E)** PA amplitude of free ICG, EV(ICG/PTX), SBC-EV(ICG/PTX) at different ICG concentrations. **(F)** Change in the size of SBC-EV(ICG/PTX) under physiological (i.e., pH 7.4) and acidic (i.e., pH 6.0) conditions, determined by DLS. After 48 h of incubation in pH 6.0 buffers, SBC-EV(ICG/PTX) were destabilized and swollen, and the size distribution increased drastically. **(G)** TEM images of SBC-EV(ICG/PTX) after incubation in pH 7.4 and 6.0 buffers for 48 h. Scale bars are 50 nm. **(H)** PTX release profiles from SBC-EV(ICG/PTX) at different pH and US (1 min of irradiation) conditions. **(I)** PTX release profiles of EV(ICG/PTX) and SBC-EV(ICG/PTX) at different pH conditions after 8 h of incubation (*p < 0.05, **p < 0.01, *n* = 3).

The loading of ICG and PTX into EVs was confirmed by UV-Vis spectroscopy. The UV-Vis absorption spectra of blank EV, ICG, PTX, ICG-loaded EV [EV(ICG)], ICG- and PTX-loaded EV [EV(ICG/PTX)], and SBC-EV(ICG/PTX) are illustrated in Figure 2A. Both EV(ICG) and EV(ICG/PTX) indicated the distinct absorption peak of ICG at 786 nm (Figure 2A). The encapsulation efficiencies of ICG and PTX for SBC-EV(ICG/PTX) were 42.9% and 39.9%, respectively (Table 1). The loading capacities of ICG and PTX for SBC-EV(ICG/PTX) were 4.76 × 10^-10^ and 1.92 × 10^-10^ μg/EVs, respectively. The loading capacities of ICG and PTX in EV(ICG/PTX) were 4.77 × 10^-10^ and 1.90 × 10^-10^ μg/EVs. This indicates that SBC does not affect the drug loading of ICG and PTX into EVs. Because PTX is a hydrophobic compound, it might be incorporated into the hydrophobic lipid bilayers of EVs, as reported in a previous study 47. Relatively hydrophilic ICG might be entrapped within the aqueous core or at the bilayer interface 48.

Because EVs are surrounded by a lipid bilayer forming hydrophilic core-hydrophobic shell structures, they can carry various hydrophobic and hydrophilic drugs. To demonstrate the capability of EVs to encapsulate versatile drugs, hydrophobic model drug, piperlongumine, and hydrophilic model drug, doxorubicin hydrochloride (DOX·HCl), were encapsulated into EVs in the same manner used for ICG and PTX. Both piperlongumine and DOX·HCl were successfully encapsulated into EVs with high encapsulation efficiencies (36.3±2.3% for piperlongumine and 41.1±3.2% for DOX·HCl), demonstrating that EVs are universal drug carriers.

The encapsulation efficiency and loading capacity of SBC in EVs were measured by quantifying of sodium ions via inductively coupled plasma optical emission spectrometry (ICP-OES). The encapsulation efficiency of SBC for SBC-EV(ICG/PTX) was 26.4%. The loading capacity of SBC for SBC-EV(ICG/PTX) was 1.09 × 10^-10^ μg/EVs. It was determined that approximately 91% of the initial amount of SBC in the SBC-EV(ICG/PTX) remained stable for about 48 h upon incubation in pH 7.4 buffers.

The sizes and surface charges of various EV-derived samples were measured. As indicated in Table 2, the sizes of blank EV, EV(ICG), SBC- and ICG-loaded EV [SBC-EV(ICG)], EV(ICG/PTX), and SBC-EV(ICG/PTX) were 117.1±2.0, 129.6±3.1, 132.7±6.8, 149.9±5.2 and 150.8±4.2 nm, respectively. The size of the EVs increased slightly when they were loaded with ICG and PTX. The zeta potentials of blank EV, EV(ICG), SBC-EV(ICG), EV(ICG/PTX), and SBC-EV(ICG/PTX) were -19.6±1.2, -20.1±0.3, -23.6±2.5, -22.7±1.2, and -26.4±2.4 mV, respectively (Table 2). The morphologies of the blank EV and SBC-EV(ICG/PTX) were analyzed by transmission electron microscopy (TEM). They exhibited spherical morphologies with diameters of 50-90 nm (Figure 2B).

The expressions of three typical EV marker proteins, CD81, CD63, and syntenin, were visualized by western blotting to verify whether the ICG- and PTX-encapsulation processes affect the protein content of EVs. Blank EV, EV(ICG/PTX), and SBC-EV(ICG/PTX) showed clear bands representing CD81, CD63, and syntenin (Figure 2C). This result indicates that the original protein content of the EVs was not affected by the drug-loading processes.

### High colloidal stability and storage stability of engineered EVs

The interaction of nanoparticles with proteins in the blood is one of the crucial factors influencing their physicochemical properties, colloidal stability, and delivery efficacy *in vivo* 49. To investigate the colloidal stability of EV(ICG/PTX) and SBC-EV(ICG/PTX) against serum proteins, they were suspended in PBS containing 10% fetal bovine serum (FBS), followed by incubation at 37 °C for 96 h. The sizes of the EV(ICG/PTX) and SBC-EV(ICG/PTX) were monitored to determine whether aggregation occurred during the incubation. As illustrated in Figure S1, both EV(ICG/PTX) and SBC-EV(ICG/PTX) did not exhibit significant size changes up to 96 h, regardless of incubation conditions, which indicates their high stability against serum proteins. The high colloidal stability of SBC-EV(ICG/PTX) ensures prolonged blood circulation for efficient chemo-SDT *in vivo*.

### Enhanced aqueous stability of ICG via encapsulation into EVs

ICG suffers from irreversible degradation by ions and radicals in aqueous solutions, reducing its absorption and fluorescence simultaneously 50, 51. EVs contain biocompatible phospholipid bilayers to protect the encapsulated cargos from extracellular environments. To investigate whether EVs can protect ICG from degradation in aqueous solutions, we quantified the fluorescence intensities of free ICG, EV(ICG), SBC-EV(ICG), and SBC-EV(ICG/PTX) during incubation in PBS at 4 °C. As represented in Figure 2D, all ICG-loaded EVs retained initial fluorescence intensities of ICG higher than 84% after 14 days of incubation. In contrast, the free ICG solution substantially lost its fluorescence after 14 days of incubation (49% of its initial value), verifying its poor aqueous stability. The findings imply that EVs can efficiently protect the entrapped ICG from destructive species in buffer solutions, confirmed by previous studies 52, 53. In addition to the high colloidal stability against serum proteins (Figure S1), the improved aqueous stability of ICG-loaded EVs indicates that EV-mediated ICG delivery exhibits substantial potential for clinical applications.

### *In vitro* PA imaging

To assess the PA properties of ICG-encapsulated EVs, the PA signals of free ICG, EV(ICG/PTX), and SBC-EV(ICG/PTX) were measured at different ICG concentrations (Figure 2E). The PA signals from all the samples at 780 nm linearly increased as the concentration increased, thus validating their excellent PA contrast.

### Acid-triggered CO_2_ generation by SBC-loaded EVs

The generation of CO_2_ from SBC-loaded EVs under acidic conditions was determined by an acid-base titration method using a commercially available CO_2_ quantification kit. CO_2_ generated from SBC-loaded EVs dissolves in aqueous solutions and forms carbonic acid (H_2_CO_3_). The carbonic acid solution was titrated with NaOH using phenolphthalein as the indicator. The relative levels of CO_2_ generation from SBC-loaded EVs and blank EVs at different pH conditions was determined by the amount of NaOH used to neutralize the solution of carbonic acid. As illustrated in Figure S2, blank EV exhibited no significant increase in CO_2_ generation compared to untreated groups, regardless of pH conditions. In contrast, SBC-EV showed a significant increase in CO_2_ generation at pH 6.0, demonstrating the acid-triggered CO_2_ generation by SBC inside EVs.

### pH-responsiveness of SBC-loaded EVs

To demonstrate the pH-responsiveness of SBC-loaded EVs, changes in size under acidic conditions were measured using dynamic light scattering (DLS). The average size and size distribution of SBC-EV(ICG/PTX) dramatically increased after 48 h of incubation in pH 6.0 buffers (Figure 2F). This result indicates that SBC-EV(ICG/PTX) are destabilized under acidic conditions, resulting from the CO_2_-triggered bursting of the EV membranes. The morphology change of the SBC-EV(ICG/PTX) under acidic conditions was observed by TEM. The TEM image clearly showed noticeable membrane destruction with pores when the SBC-EV(ICG/PTX) were incubated in pH 6.0 buffers (Figure 2G). This result demonstrates the acid-triggered destabilization of the SBC-EV(ICG/PTX). Therefore, it is expected that SBC-EV(ICG/PTX) would facilitate drug release under acidic conditions.

### Dual pH- and US-responsive drug release by SBC-loaded EVs

To validate the ability of SBC-loaded EVs to realize pH-responsive drug release, the release profiles of PTX and ICG were determined at different pH conditions via the dialysis method. In addition, to evaluate whether US could facilitate pH-sensitive drug release from the SBC-loaded EVs, the release of PTX was determined at different pH conditions (e.g., endo/lysosomal pH of 6.0 and tumor microenvironmental pH of 6.6) in the presence or absence of US irradiation. As represented in Figure 2H, the cumulative release of PTX at pH 5.0 and 6.0 was markedly higher than that at pH 7.4, thereby demonstrating that pH-sensitive SBC-EV(ICG/PTX) can rapidly release PTX under mildly acidic conditions, similar to that of endo/lysosomal compartments. Interestingly, regardless of pH conditions, US treatment further facilitated the release of PTX, demonstrating the dual pH- and US-responsive drug release by SBC-loaded EVs (Figure 2H). The US-triggered drug release by SBC-loaded EVs can be ascribed to the US-induced cavitation effect (collapse of vapor-filled bubbles in liquids). This collapse creates shock waves that can destabilize SBC-EV(ICG/PTX) and release the encapsulated drugs 54. In addition, US irradiation-triggered ROS generation from ICG may lead to lipid peroxidation and consequent destabilization of EVs, facilitating the release of ICG and PTX 55. This physical and chemical disruption of EVs through US irradiation would facilitate the release of PTX entrapped in the hydrophobic lipid bilayers of EVs.

The cumulative release of PTX from SBC-EV(ICG/PTX) was the highest when incubated at pH 5.0, in the presence of US (Figure 2H). To verify that SBC is an actual contributor in triggering the pH-sensitive drug release from SBC-loaded EVs, we compared the cumulative PTX release from EV(ICG/PTX) and SBC-EV(ICG/PTX) at different pH conditions. The amount of PTX released from SBC-EV(ICG/PTX) was significantly higher than that from EV(ICG/PTX) when they were incubated at pH 5.0 (Figure 2I). In contrast, the amount of PTX released from SBC-EV(ICG/PTX) was similar to that released from EV(ICG/PTX) when incubated at pH 7.4, thereby implying that SBC primarily contributes to the pH-sensitive drug release of the SBC-EV(ICG/PTX). The dual pH/US-dependent ICG release from SBC-EV(ICG/PTX) was also demonstrated (Figure S3). The highest cumulative release of ICG was observed at pH 5.0, regardless of US irradiation and incubation time (Figure S3). Similar to what was observed with the PTX release, the release of ICG from SBC-EV(ICG/PTX) was further facilitated by US treatment. Drug release profiles of SBC-EV(ICG/PTX) were also evaluated at pH 6.6 that mimics mildly acidic tumor microenvironment conditions. As shown in Figure S4, the amounts of PTX and ICG released from SBC-EV(ICG/PTX) were significantly higher than those from EV(ICG/PTX) when they were incubated at pH 6.6. The dual pH- and US-responsive SBC-loaded EVs would regulate the release of drugs more precisely at the target tumor region. The enhanced release of ICG and PTX by dual pH/US-responsive EVs would ensure high anticancer effects of chemo-SDT in target cancer cells.

### Efficient cellular uptake of ICG-loaded EVs

**Figure 3:**
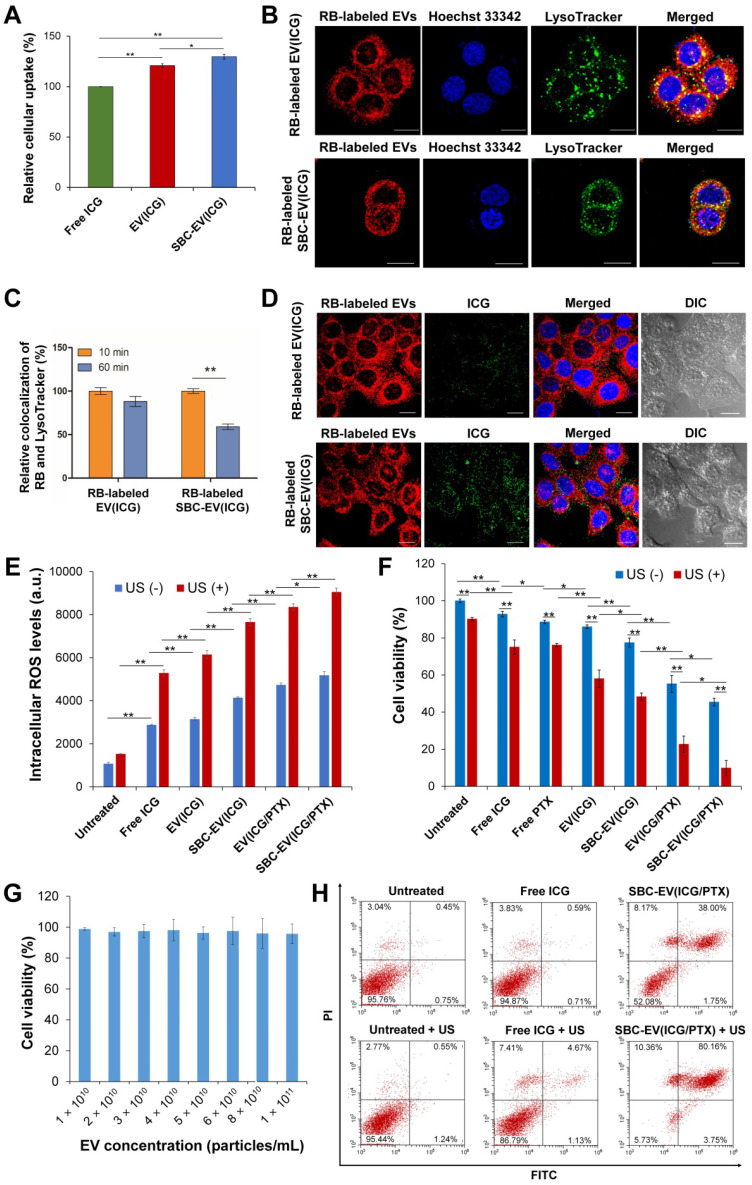
** (A)** Relative internalization of ICG by MCF-7 cells after 4 h of treatment with free ICG, EV(ICG), and SBC-EV(ICG). **(B)** Live confocal microscopic images of MCF-7 cells treated with RB-labeled EV(ICG) and RB-labeled SBC-EV(ICG). LysoTracker (green dots) was used to stain endo/lysosomal compartments. Hoechst 33342 (blue dots) was used to stain cellular nuclei. Scale bar = 20 µm. **(C)** Relative colocalization ratios of RB with LysoTracker in MCF-7 cells after incubation with RB-labeled EV(ICG) and RB-labeled SBC-EV(ICG) for different incubation times. **(D)** Intracellular distribution of RB-labeled EVs (red dots) and ICG (green dots) in live MCF-7 cells, observed by confocal laser scanning microscopy. The cells were treated with RB-labeled EV(ICG) and RB-labeled SBC-EV(ICG) for 1 h. Differential interference contrast images show the cell morphology throughout the imaging process. Scale bar = 20 µm. **(E)** Intracellular ROS levels in MCF-7 cells treated with free ICG and ICG-loaded EV samples before and after US treatment. **(F)** Viabilities of MCF-7 cells after treatment with various EV samples before and after US irradiation (0.3 W/cm^2^ for 1 min). **(G)** Viabilities of MCF-7 cells treated with blank EVs at varying concentrations. Data were represented as mean ± SD (*p < 0.05, **p < 0.01, *n* = 3). **(H)** Apoptosis analysis of MCF-7 cells treated with free ICG and SBC-EV(ICG/PTX) before and after US treatment.

To investigate whether EVs can enhance intracellular delivery of their therapeutic cargos, free ICG, EV(ICG), and SBC-EV(ICG) at equivalent concentrations of ICG were added to MCF-7 human breast cancer cells. The cellular uptake of ICG for EV(ICG) and SBC-EV(ICG) was significantly increased compared to free ICG (Figure 3A). The enhanced cellular uptake of EV(ICG) and SBC-EV(ICG) can be explained by the favorable interaction of EVs with the cell membranes via receptor-ligand binding or direct fusion 56. It is well-documented that EVs efficiently enter the cells via numerous molecular mechanisms, including clathrin-dependent receptor-mediated endocytosis, micropinocytosis, membrane fusion, etc 57. The routes of cellular uptake depend on the presence of specific surface proteins on both the EVs and the recipient cells. Although it is still unclear which uptake mechanism is preferred, EVs can fuse with endosomal/lysosomal membranes and release their cargos into the cytoplasm 58.

To investigate whether SBC in EVs affects their cellular uptake, fluorescein isothiocyanate (FITC)-labeled EVs were used to prepare EV(ICG) and SBC-EV(ICG). The cellular uptake of SBC-EV(ICG) and EV(ICG) was compared by measuring the fluorescence intensity of FITC. The result showed that there was no significant difference in cellular uptake between FITC-EV(ICG) and FITC-SBC-EV(ICG) (Figure S5).

### Enhanced cytoplasmic release of ICG by SBC-loaded EVs

To investigate whether SBC-loaded EVs can facilitate the cytoplasmic release of their cargos, intracellular localization and endosomal escape of SBC-EV(ICG) were evaluated by confocal laser scanning microscopy. To track the intracellular distribution of EVs, rhodamine B (RB)-labeled EVs were used to encapsulate SBC and ICG. The resulting RB-labeled SBC-EV(ICG) was incubated with live MCF-7 cells. RB-labeled EV(ICG) was also used as a non-pH-responsive counterpart. MCF-7 cells were stained with LysoTracker, which selectively accumulates into endo/lysosomal regions, to investigate whether EVs can be internalized by the cells via endocytosis. As shown in Figure 3B, a large amount of RB-labeled EV(ICG) and RB-labeled SBC-EV(ICG) (red dots) was colocalized with LysoTracker (green). This result confirms that ICG-loaded EVs were internalized by the cells via endocytosis, which is consistent with previous studies 59.

After endocytosis of EVs, a prerequisite for efficient cytoplasmic delivery of their cargos is their escape from endo/lysosomal compartments. To investigate whether ICG-laded EVs can escape the endo/lysosomal compartments, the degree of colocalization between RB-labeled EVs and Lysotracker was quantified at different incubation times. The quantitative result (Figure 3C) revealed that colocalization of pH-responsive RB-labeled SBC-EV(ICG) with endo/lysosomes significantly decreased with time. In contrast, colocalization of non-pH-responsive RB-labeled EV(ICG) with endo/lysosomes was slightly reduced. The more efficient endosomal escape of the SBC-EV(ICG) might be implicated in the destabilization of endo/lysosomal membranes via bursting effects of the SBC-EV(ICG) in response to acidic endo/lysosomal pH. However, the detailed mechanism of endosomal escape of the SBC-loaded EVs still remained unexplored. A recent study proposed that direct fusion of EVs with endo/lysosomal membranes is one the main mechanisms for endo/lysosomal escape of EVs 59. This study demonstrated that a fraction of internalized EVs undergo fusion with endo/lysosomal membranes, resulting in the release of their cargos 59. The fusion of EVs with the endo/lysosomal membranes is facilitated by the acidic pH in the endo/lysosomes that may induce changes in membrane fluidity and lipid compositions of EVs 60.

Efficient cytoplasmic release of therapeutic cargos from drug carriers is crucial for maximizing their therapeutic efficacies. Subcellular distribution of RB-labeled EVs (red dots) and ICG (green dots) after 1 h of incubation with RB-labeled EV(ICG) and RB-labeled SBC-EV(ICG) are shown in Figure 3D. While RB-labeled EV(ICG)-treated cells exhibited a weak yellow fluorescence signal, RB-labeled SBC-EV(ICG)-treated cells showed a strong yellow fluorescence signal. Because aggregated ICG induces self-quenching in fluorescence, the weak fluorescence of ICG from the RB-labeled EV(ICG)-treated cells indicates that ICG is mainly accumulated inside the EVs. However, pH-sensitive SBC-EV(ICG) rapidly released the encapsulated ICG via acid-destabilization of the EVs in the endo/lysosomal compartments, thereby leading to the increased fluorescence intensity of ICG (Figure 3D). This result demonstrates that pH-responsive SBC-loaded EVs achieve efficient cytoplasmic release of encapsulated cargos, which would enhance their combination chemo-SDT efficacies.

### US-triggered intracellular ROS generation by ICG-loaded EVs

Cancer cells are in a state of redox imbalance because they possess higher intracellular ROS levels than normal cells. Consequently, the excessive accumulation of ROS in cancer cells induces cell death 2. Intracellular ROS concentrations in MCF-7 cells after incubation with ICG, EV(ICG), SBC-EV(ICG), or SBC-EV(ICG/PTX) were quantified using ROS-sensing dyes. In addition, the cells were exposed to US irradiation to trigger sonodynamic effects. As shown in Figure 3E, regardless of US irradiation, the treatment of free ICG (28 µg/mL) and ICG-loaded EVs (28 µg/mL ICG) increased the intracellular ROS levels in the cells. It was reported that US irradiation in liquid environments generates cavitating bubbles 61, 62. Sonoluminescent light created by the implosion of cavitating bubbles can excite organic sonosensitizers, such as ICG. Consequently, ROS is generated from the excited sonosensitizers, similar to the photodynamic effects 61, 62.

Regardless of US irradiation, the cells treated with EV(ICG) exhibited higher intracellular ROS levels than those treated with free ICG (Figure 3E). This result is closely associated with the enhanced stability and cellular uptake of EV(ICG) in comparison with free ICG. EV(ICG/PTX) induced higher levels of intracellular ROS in the cells than did EV(ICG), regardless of US irradiation. PTX has been reported to promote ROS generation via the elevated activity of nicotinamide adenine dinucleotide phosphate (NADPH) oxidase associated with plasma membranes 63. To demonstrate that PTX elevates intracellular ROS levels, the relative mRNA expression level of NADPH oxidase 1 (NOX1) in MCF-7 cells was analyzed after the treatment with PTX. After 2 h of incubation with PTX, the relative mRNA expression level of NOX1 in MCF-7 cells was significantly increased (Figure S6). Together with free ICG, all ICG-loaded EVs drastically increased intracellular ROS levels in response to the US. SBC-EV(ICG/PTX) combined with the US exhibited the highest intracellular ROS levels. US-triggered ROS generation by SBC-EV(ICG/PTX) was visualized by fluorescence images of 2',7'-dichlorofluorescein diacetate (DCF-DA)-stained MCF-7 cells (Figure S7). The cells incubated with free ICG revealed weak green fluorescence upon US irradiation. In contrast, the cells treated with SBC-EV(ICG/PTX) showed strong green fluorescence upon US exposure (Figure S7).

To investigate whether SBC in the SBC-EV(ICG/PTX) plays a role in the increase in intracellular ROS, we assessed the intracellular ROS production of MCF-7 cells treated with 4 nM SBC, which is the equivalent concentration used for SBC-EV(ICG/PTX). As illustrated in Figure S8, regardless of US irradiation, SBC increased intracellular ROS, but the increase was relatively small compared to free ICG (28 µg/mL). The treatment with free PTX (10 µg/mL) also increased intracellular ROS in MCF-7 cells. To investigate whether 4 nM SBC can induce cytotoxicity, cytotoxicity of SBC-EV against MCF-7 cells was determined before and after US irradiation. As shown in Figure S9, SBC-EV containing 4 nM SBC did not exhibit high cytotoxicity, regardless of US irradiation (~96% viability before US irradiation and ~85% viability after US irradiation). It is known that increased intracellular ROS can induce cell death when it exceeds a cytotoxic threshold level 1. The increase in intracellular ROS levels by SBC-EV might not be sufficient to cause significant cytotoxicity.

### Optimization of drug encapsulation ratios of SBC-EV(ICG/PTX) and US irradiation intensity for efficient chemo-SDT combination therapy

To determine the optimized encapsulation ratio of SBC in SBC-EV(ICG/PTX) for efficient chemo-SDT combination therapy, cytotoxicity of SBC-EV(ICG/PTX) at different concentrations of SBC was evaluated using MCF-7 cells. As shown in Figure S10, high SBC concentrations in EVs resulted in some cytotoxicity against MCF-7 cells. The SBC concentration in SBC-EV(ICG/PTX) was set to 4 nM because higher concentrations of SBC than 4 nM led to considerable cytotoxicity.

Cytotoxicity of SBC-EV(ICG/PTX) at different encapsulation ratios of ICG and PTX was evaluated using MCF-7 cells to optimize the encapsulation ratios of ICG and PTX for efficient chemo-sonodynamic therapeutic effects. The half maximal inhibitory concentration (IC_50_) values of ICG and PTX in each SBC-EV(ICG/PTX) formulation were determined by the cell viability results at various concentrations of ICG and PTX. As displayed in Figure S11, regardless of US treatment, increasing the encapsulation ratio of PTX in SBC-EV(ICG/PTX) led to the increased cytotoxicity. The cytotoxicity of SBC-EV(ICG/PTX) was significantly increased upon US irradiation when 28 µg/mL ICG and 10 µg/mL PTX were loaded into the EVs. Because very high concentrations of PTX may induce severe cytotoxicity against normal cells even in the absence of US, the optimized PTX concentration was determined to be 10 µg/mL. The IC_50_ values of various SBC-EV(ICG/PTX) samples before and after US irradiation were represented in Table S1. The IC_50_ values of ICG and PTX in SBC-EV(ICG/PTX) were decreased as the encapsulation ratios of PTX in SBC-EV(ICG/PTX) increased, regardless of US irradiation. The IC_50_ values of ICG and PTX in SBC-EV(ICG/PTX) were noticeably dropped when the cells were treated with US irradiation, demonstrating the effective chemo-SDT by SBC-EV(ICG/PTX). The IC_50_ values of ICG and PTX in SBC-EV(ICG/PTX) encapsulating 28 µg/mL ICG and 10 µg/mL PTX under US irradiation were found to be 17.17 and 6.13 µg/mL, respectively.

To investigate whether different power densities of US influence anticancer activity of SBC-EV(ICG/PTX), cell viability of MCF-7 cells treated with SBC-EV(ICG/PTX) was evaluated after irradiation with US at different power densities. As shown in Figure S12, the cell viability was decreased as the US power intensities increased. It should be noted that US irradiation itself leads to some cytotoxicity against MCF-7 cells depending on its power density. Therefore, this study chose 0.3 W/cm^2^ (*in vitro*) or 0.5 W/cm^2^ (*in vivo*) of US power density to minimize the cytotoxic effect by US irradiation itself.

### *In vitro* chemo-SDT efficacy of SBC-EV(ICG/PTX)

US-triggered chemo-sonodynamic anticancer activity of SBC-EV(ICG/PTX) against human breast cancer cells was investigated. MCF-7 cells received the treatment of free ICG, EV(ICG), SBC-EV(ICG), EV(ICG/PTX), and SBC-EV(ICG/PTX), followed by 1 min of US irradiation (1 MHz, 0.3 W/cm^2^). As represented in Figure 3F, the cytotoxicity of US irradiation was minimal to the cells. More than 90% of the cells retained their viability after US irradiation only. Treatment with free ICG and ICG-loaded EVs without US treatment exhibited some cytotoxicity. MCF-7 cells treated with EV(ICG) exhibited lower viability than those incubated with free ICG, which is closely associated with the enhanced ICG uptake by EVs (Figure 3A and F). When the cells received a combination of ICG-loaded EVs and US irradiation, a substantial drop in cell viability was found owing to the sonodynamic effects of ICG. For example, the viabilities of the cells that received free ICG and EV(ICG) with US irradiation were 75.1% and 58.1%, respectively (Figure 3F). EV(ICG/PTX) significantly decreased the cell viability from 55.3% to 22.7% under US irradiation owing to the combinatorial effect of SDT and chemotherapy. Notably, the pH-sensitive SBC-EV(ICG/PTX) revealed significantly increased cytotoxicity against MCF-7 cells than EV(ICG/PTX), regardless of US treatment. This result can be explained by the efficient release of PTX from the EVs in response to the acidic pH in the endo/lysosomes (Figure 2I). Significantly high cytotoxicity (9.9% cell viability) was observed when the cells received SBC-EV(ICG/PTX), followed by US irradiation. The facilitated release of ICG and PTX from the EVs in the acidic endo/lysosomal compartments might contribute to the high anticancer effects of the combination chemo-SDT (Figure 2H, Figure S3, and Figure 3D). These results also demonstrate that EVs are suitable nanocarriers for the efficient intracellular transport of hydrophilic ICG and hydrophobic PTX, simultaneously, which results in effective combined chemo-SDT.

The cytotoxicity of blank EVs in MCF-7 cells was also examined to demonstrate their high biosafety. The blank EVs showed negligible cytotoxicity (> 95% viability) up to a 1.0 × 10^11^ particles/mL concentration (Figure 3G). This result clearly verifies that EVs are safe nanocarriers. Because the intrinsic toxicity of drug carriers is a significant limitation to clinical translations, the negligibly low toxicity of EVs demonstrates their substantial potential for successful clinical use.

To investigate whether the US-triggered sonodynamic effects of SBC-EV(ICG/PTX) induced apoptosis, we quantified the apoptotic MCF-7 cells treated with free ICG and SBC-EV(ICG/PTX) before and after US irradiation. Annexin V-FITC/PI staining results indicate a substantial increase in the population of total apoptotic cells (early apoptosis + late apoptosis) when the cells received free ICG and SBC-EV(ICG/PTX) plus US treatment (Figure 3H). Treatment with SBC-EV(ICG/PTX) combined with US irradiation significantly increased the percentage of total apoptotic cells from 39.75% to 83.91%. The apoptosis results were consistent with cytotoxicity results, as illustrated in Figure 3F. Combined with the analysis of intracellular ROS generation (Figure 3E), the apoptosis results indicate that the US-triggered sonodynamic effects of SBC-EV(ICG/PTX) induce the apoptosis of cancer cells through elevated oxidative stress.

Sonotoxicities of free ICG and SBC-EV(ICG/PTX) against MCF-7 cells were visualized via calcein acetoxymethyl ester (calcein AM, green, live cells)/ethidium homodimer-1 (EthD1, red, dead cells) co-staining. Fluorescence images of the co-stained MCF-7 cells after various treatments are illustrated in Figure S13. The cells treated with SBC-EV(ICG/PTX) and US irradiation showed the lowest viability, which was consistent with the cytotoxicity results (Figure 3F and 3H).

### Effective tumor accumulation of SBC-EV(ICG/PTX)

**Figure 4:**
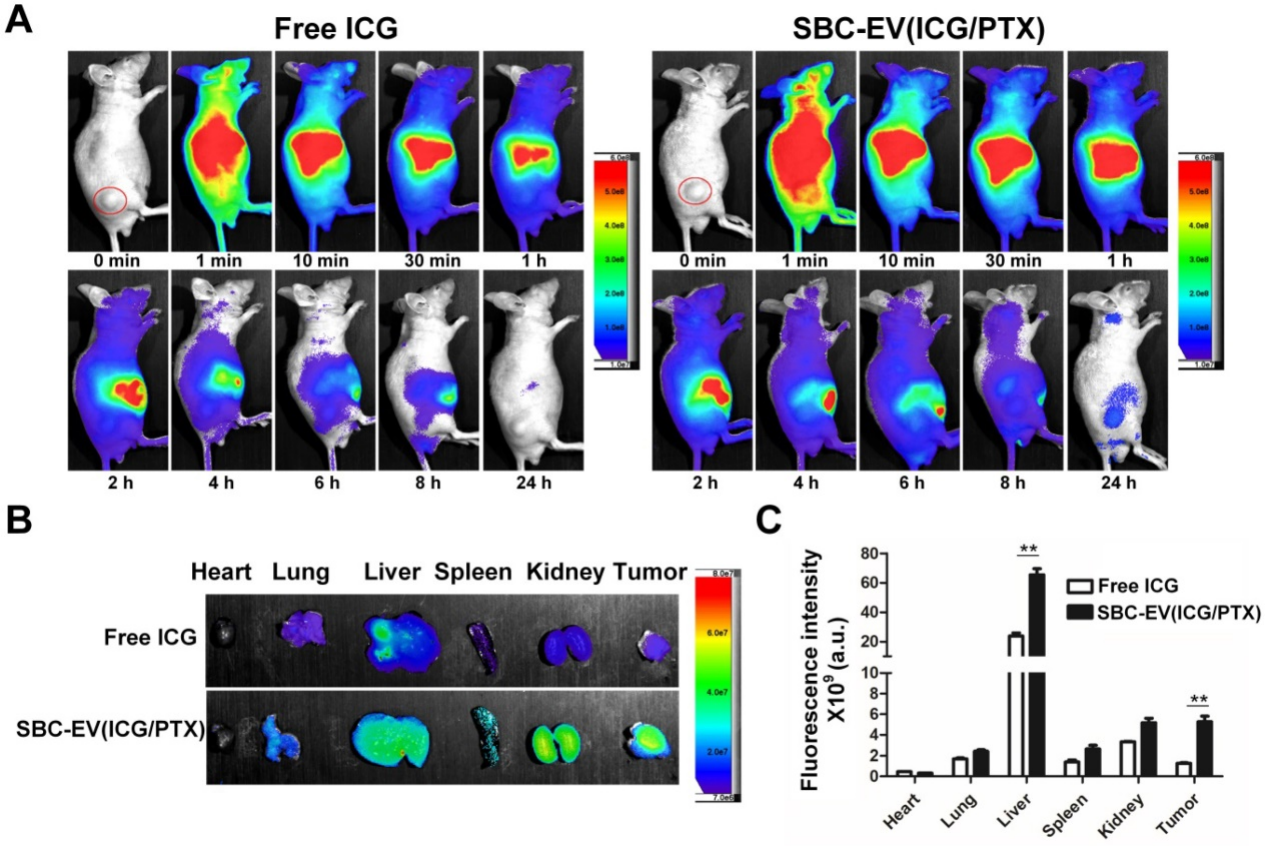
** (A)** *In vivo* biodistribution images from tumor-xenograft mice after i.v. administration of free ICG and SBC-EV(ICG/PTX). **(B)** *Ex vivo* imaging of tumors and major organs at 24 h-post injection of free ICG and SBC-EV(ICG/PTX). **(C)** Quantification of ICG from *ex vivo* imaging of major organs at 24 h-post i.v. administration of free ICG and SBC-EV(ICG/PTX) (**p < 0.01, *n* = 4).

It is well-documented that nanocarriers, such as the EVs in this study, leverage the EPR effect for facilitated extravasation into the tumors 64. EVs' preferential tumor accumulation of ICG was verified via *in vivo* real-time fluorescence imaging in mice bearing MCF-7 xenografts (Figure 4A). The biodistribution of free ICG and SBC-EV(ICG/PTX) in the mice at different time intervals (0-24 h post-injection) was visualized by using an *in vivo* imaging system (IVIS), which can quantify the NIR fluorescence intensity of the ICG. The tumor of the mice treated with SBC-EV(ICG/PTX) showed fluorescence until 24 h post-injection (Figure 4A). On the contrary, the fluorescence in the tumor of the free ICG-treated mice almost disappeared at 24 h after injection. These findings confirm that ICG was efficiently delivered into tumors via the EPR effect of nanoscale EVs. The passive tumor targeting of EVs has been reported elsewhere 65.

We assessed the distribution of ICG in the entire organs of the tumor-bearing mice after the intravenous (i.v.) administration of SBC-EV(ICG/PTX) and free ICG. The* ex vivo* imaging of the resected tumors and major organs revealed the biodistribution of SBC-EV(ICG/PTX) and free ICG at 24 h after injection (Figures 4B). Both SBC-EV(ICG/PTX)-treated mice and free ICG-treated mice exhibited the highest level of ICG in the liver at 24 h post-injection (Figures 4B and 4C). Efficient tumor accumulation of SBC-EV(ICG/PTX) was also confirmed. The level of ICG localization in the tumors of the SBC-EV(ICG/PTX)-treated mice at 24 h post-administration was markedly higher than that for the free ICG-treated mice (Figures 4B and 4C), which corresponded with the biodistribution results (Figure 4A). This result indicates that EVs can accumulate into tumors more efficiently than free ICG. Quantification of ICG from *ex vivo* imaging of liver and tumor at 4 h and 24 h-post i.v. administration of free ICG and SBC-EV(ICG/PTX) was also carried out. As represented in Figure S14, accumulation of free ICG and SBC-EV(ICG/PTX) in tumor and liver of mice was significantly higher at 4 h post-injection than that at 24 post-injection.

One of the main biological functions of EVs is prolonged blood circulation because they can avoid the clearance by the mononuclear phagocyte system 66. To demonstrate EVs' prolonged blood circulation time, the concentration-time profiles of free ICG and EV(ICG/PTX) in plasma of tumor-bearing mice were determined after intravenous administration. The area under the plasma concentration-time curve (AUC) of ICG from tumor-bearing mice over a 24 h-period determined for the EV(ICG/PTX) groups increased significantly compared to that for free ICG groups (Table S2). The long-term blood circulatory capability of EV(ICG/PTX) can be attributed to the endogenous origin and specific membrane proteins of the EVs 66. A previous study using ICG-loaded liposomes showed only ~1.8-fold higher AUC of ICG over a 24 h-period compared to free ICG 67.

Because ionic compounds such as SBC can diffuse outside the EVs depending on the concentration gradient, we investigated the stability of SBC in the EVs under *in vivo*-mimicking conditions. Release profiles of SBC from EVs were determined at high salt concentrations (i.e., 260 mM KCl aqueous solution) that mimic *in vivo* conditions 68. The released amount of SBC from EVs using the dialysis method was measured by the quantification of sodium ions via ICP-OES. It was found that 91.3% of SBC was maintained in the EVs up to 48 h of incubation against the high salt concentration (Figure S15). The slow release of SBC from EVs might be ascribed to the low ion permeability of the phospholipid bilayers. Considering the fact that a large amount of SBC-EV(ICG/PTX) accumulated into tumors of mice within 24 h after injection (Figure 4A-4C), SBC remained in the EVs could trigger pH-sensitive drug release in response to acidic pH in the endo/lysosomes after endocytosis.

### *In vivo* PA imaging using ICG-loaded EVs

**Figure 5:**
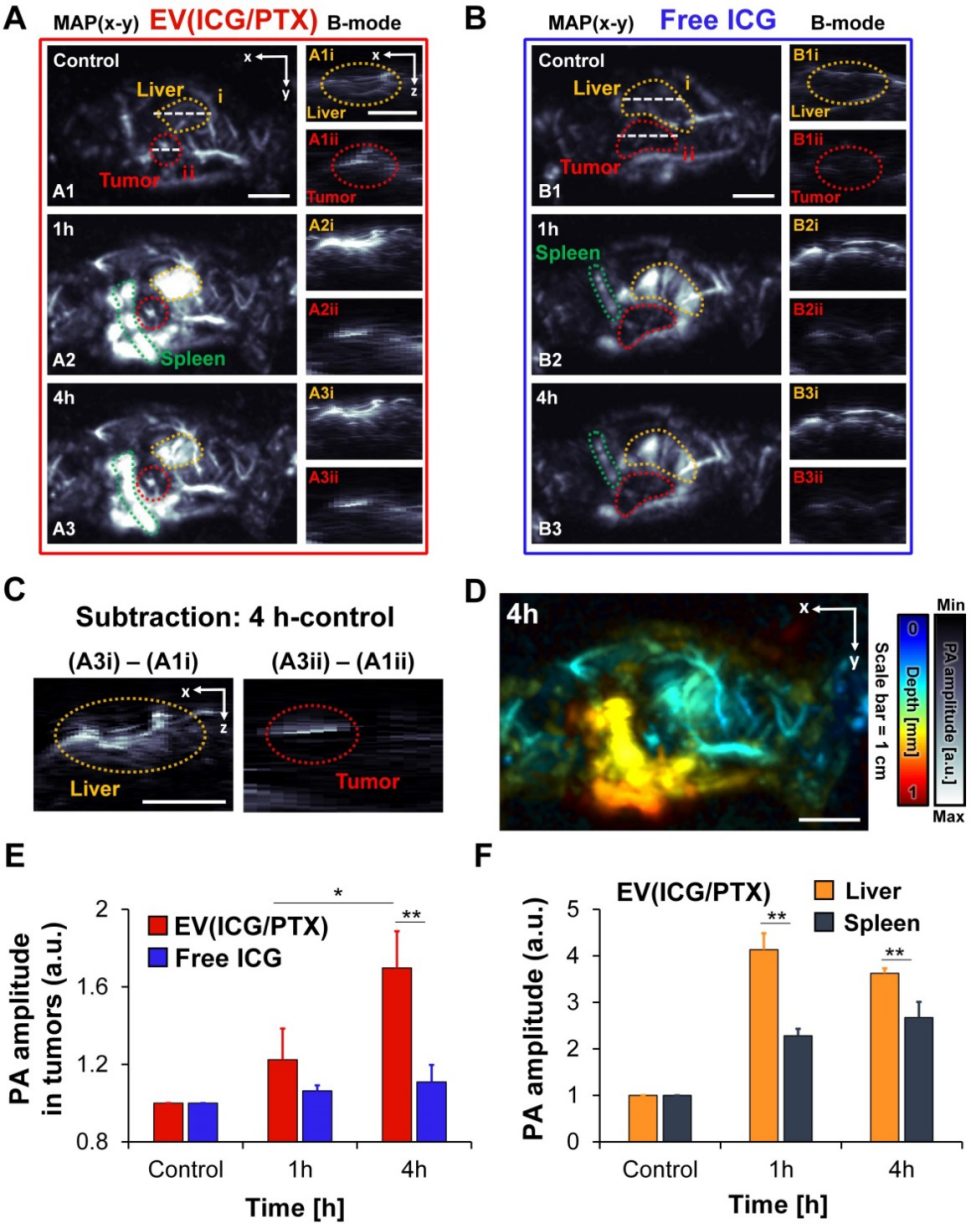
***In vivo* whole-body PA images of MCF-7-bearing mice at different time intervals after tail vein injection of EV(ICG/PTX) and free ICG. (A)** PA MAP and depth-resolved B-mode images of a mouse after i.v. injection of EV(ICG/PTX) (MAP: maximum amplitude projection). Scale bar indicates 1 cm. **(B)** PA MAP and depth-resolved B-mode images of the mouse after administration of free ICG. Scale bar indicates 1 cm. **(C)** Subtraction of PA images (4 h - control) of tumor and liver. Scale bar indicates 1 cm. **(D)** Depth-coded MAP PA image at 4 h after i.v. injection of EV(ICG/PTX). Scale bar indicates 1 cm. **(E)** Quantified PA amplitude enhancement in tumor region after injection of EV(ICG/PTX) and free ICG. **(F)** Quantified PA amplitude enhancement in organs (liver and spleen) after injection of EV(ICG/PTX) (*p < 0.05, **p < 0.01).

Because ICG exhibits the high absorption in the NIR region, ICG-loaded EVs can serve as PA contrast agents. *In vivo* PA imaging of ICG-loaded EVs and free ICG was performed using MCF-7 tumor-bearing mice. The whole-body PA maximum amplitude projection (MAP) images and cross-sectional PA B-mode images of mice treated with EV(ICG/PTX) and free ICG are presented in Figures 5A and B, respectively. Strong PA signals were observed in the tumor region time-dependently when the mice were treated with EV(ICG/PTX) (Figure 5A). The accumulation of EV(ICG/PTX) within the tumor and liver was also visualized in the depth-resolved PA B-mode images (Figures 5A1-A3). In contrast, over time, no strong PA signals were identified in the tumor areas when the mice received free ICG (Figure 5B). The subtraction PA B-mode images (4 h - control) in the tumor and liver areas confirmed the significant accumulation of EV(ICG/PTX) (Figure 5C). A more significant increase in PA signals was observed at 4 h post-injection than that acquired at pre-injection. The depth-coded PA MAP image of the mouse treated with EV(ICG/PTX) at 4 h post-injection is presented in Figure 5D. The depth-coded PA MAP image shows that EV(ICG/PTX) is highly accumulated in the tumor and other organs (liver and spleen). Figure 5E presents a quantitative comparison of the PA signal amplitudes in the tumor areas of the mice injected with free ICG and EV(ICG/PTX). The mice injected with EV(ICG/PTX) exhibited higher PA signal intensity than those injected with free ICG. The *in vivo* biodistribution of the major organs (liver and spleen) treated with EV(ICG/PTX) was photoacoustically quantified (Figure 5F). The strongest PA signals were found in the liver at 1 h after injection of EV(ICG/PTX), and the PA signals in the liver were stronger than those in the spleen throughout 4 h after injection. This high localization of nanoparticles in the liver has been commonly observed with almost all nanoparticles used for *in vivo* drug delivery because the liver is a highly vascularized organ with leaky endothelium 69. In addition, *in vivo* PA MAP imaging of tumor of mice injected with SBC-EV(ICG/PTX) was conducted at different time intervals (1 h and 4 h) (Figure S16). Strong PA signals were observed in the tumor in a time-dependent manner. This *in vivo* biodistribution study via PA imaging demonstrates that ICG-loaded EVs can act as effective PA contrast agents *in vivo*, enabling PA imaging-guided combination chemo-SDT.

### Enhanced *in vivo* SDT by pH-sensitive SBC-EV(ICG/PTX)

**Figure 6:**
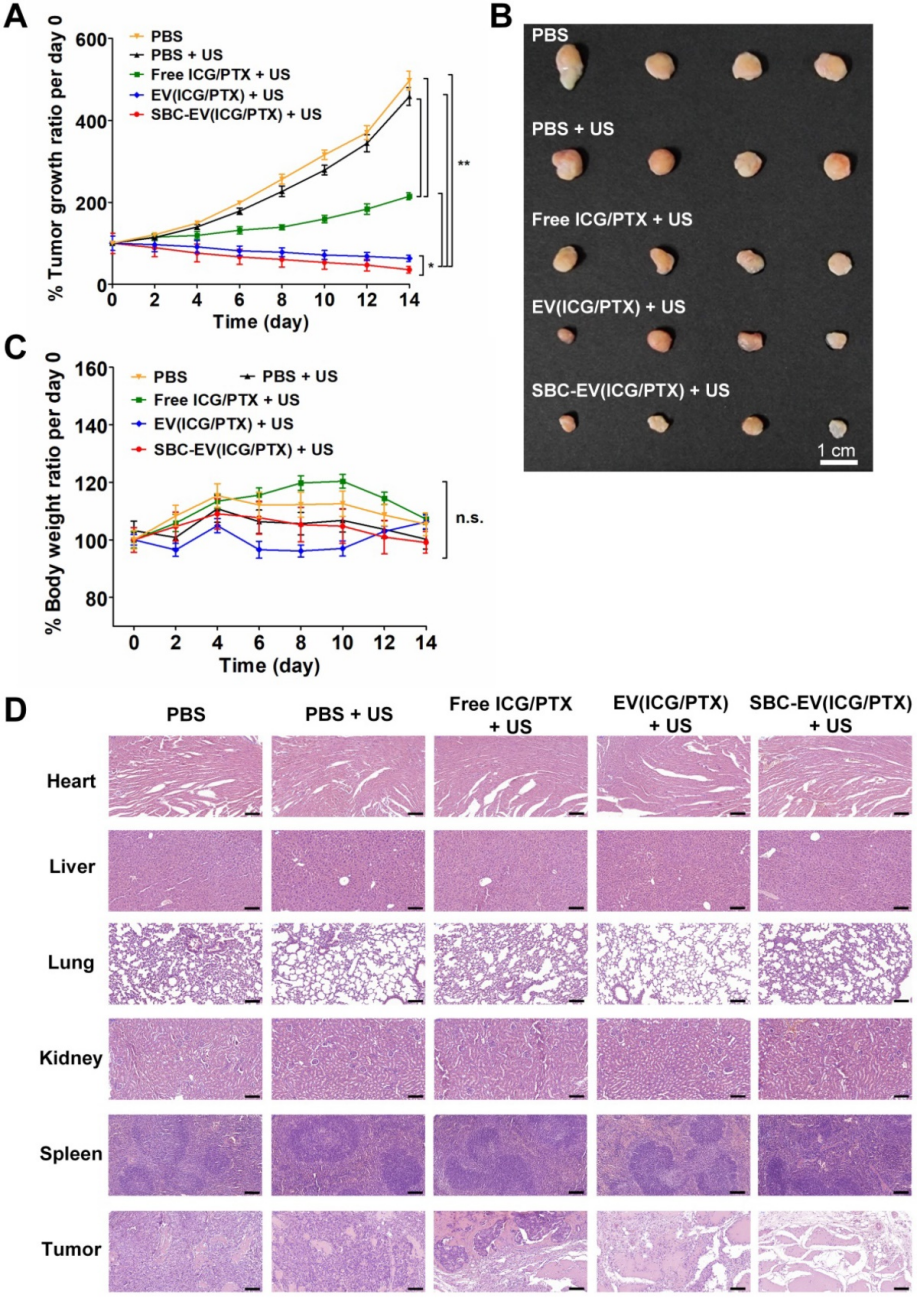
** (A)** Normailzed tumor growth ratio of MCF-7 tumor-bearing mice (*n* = 4) for 14 days after administration of various samples and US irradiation. The mice were irradiated with US at 4 h post-i.v. injection of each sample. **(B)** Representative images of tumors at day 14 after i.v. injection of samples and US irradiation. **(C)** Changes in body weights of tumor-bearing mice (*n* = 4) for 14 days after i.v. injection of various samples and US irradiation (*n* = 4) (*p < 0.05, **p < 0.01, n.s.; not significant). **(D)** H&E staining of major organs of the mice at day 14 after i.v. injection of various samples and US irradiation. Scale bar = 200 µm.

The *in vivo* SDT efficacies of various EVs were investigated using MCF-7 tumor-xenograft mouse models. Various samples [e.g., PBS, free ICG/PTX, EV(ICG/PTX), and SBC-EV(ICG/PTX)] were injected into the tail vein of the tumor-grafted mice. To find optimal time points for US irradiation, the fluorescence intensity ratios of tumor core area/peritumoral tissue area (C/P) in the SBC-EV(ICG/PTX)-treated mice were calculated. As illustrated in Figure S17, the highest C/P ratio was observed at 4 h post-injection. Therefore, the tumors were irradiated with 3 min of US (1 MHz, 0.5 W/cm^2^) at 4 h post-injection of each sample. The tumor volumes were monitored over 14 days. There was no extra injection of samples or US treatment. As illustrated in Figure 6A, treatment with EV(ICG/PTX) or SBC-EV(ICG/PTX) significantly suppressed the tumor growth more than the treatment with free ICG/PTX. Notably, SBC-EV(ICG/PTX) showed a higher efficacy in tumor growth suppression than that of EV(ICG/PTX) (Figure 6A). The tumor images of the SBC-EV(ICG/PTX)-treated mice on day 14 also indicated the most suppressed growth of tumors (Figure 6B). This indicates that the SBC-EV(ICG/PTX) induced a significant inhibition of tumor proliferation owing to the facilitated cytoplasmic release of ICG and PTX in cancer cells, which was triggered by the acidic pH in the endo/lysosomes. The body weights of all the tested groups did not show significant changes over 14 days, indicating that no adverse side effects were induced by all the treatments (Figure 6C). All the mice for each group survived up to day 14 after the treatments (survival rates = 100%). The hematoxylin and eosin (H&E) staining images revealed the severe nuclear condensation and fragmentation in the tumor of the mice treated with SBC-EV(ICG/PTX) and US (Figure 6D).

*In vivo* toxicity of SBC-EV(ICG/PTX) combined with the US was evaluated by blood biochemical analysis. Serum levels of alanine aminotransferase (ALT, indicators of hepatotoxicity), albumin (ALB, indicator of liver functions), and blood urea nitrogen (BUN, indicator of nephrotoxicity) were measured from blood of mice at 14 day post-i.v. injection of SBC-EV(ICG/PTX). As shown in Figure S18, there were no significant changes in the levels of ALT, ALB, and BUN in blood samples of the mice compared to those of control groups, indicating high biosafety of SBC-EV(ICG/PTX). Moreover, pathological changes were not found in the major organs of the EV(ICG/PTX)- and SBC-EV(ICG/PTX)-treated mice (Figure 6D). These results clearly demonstrate the high efficacy and biosafety of ICG-loaded EVs as nanosonosensitizers.

## Conclusions

We successfully developed novel dual US/pH-sensitive EV-based nanosonosensitizers for biocompatible and efficient chemo-sonodynamic combination cancer therapy. The stability of ICG in aqueous solutions was significantly improved by the encapsulation into EVs. The cellular uptake of ICG was also improved by the encapsulation into EVs, leading to a significant increase in the sonodynamic effects. Notably, SBC-EV(ICG/PTX) exhibited pH-responsive drug release owing to bursting effects triggered by the generation of CO_2_ under acidic conditions. Drug release from the SBC-EV(ICG/PTX) was further increased by US irradiation. After endocytosis, pH-responsive SBC-loaded EVs exhibited efficient cytoplasmic release of ICG in response to acidic pH in the endo/lysosomes. SBC-EV(ICG/PTX) significantly increased intracellular ROS levels upon US irradiation. Accordingly, SBC-EV(ICG/PTX) exhibited the highest sonotoxicity against human breast cancer cells. Because ICG functions as a PA imaging agent, the enhanced localization of ICG in the tumor of EV(ICG/PTX)-injected mice was visualized via high-resolution *in vivo* PA imaging. Consequently, EV(ICG/PTX) inhibited tumor growth in tumor-bearing mice more effectively than free ICG and PTX. Notably, pH-sensitive SBC-EV(ICG/PTX) with US irradiation exhibited the most efficient antitumor activity in tumor-bearing mice. This study demonstrated that SBC-EV(ICG/PTX) is a highly biocompatible and effective sonotheranostic nanoplatform for combined PA imaging and chemo-sonodynamic cancer therapy.

## Methods

### Materials

2',7'-dichlorofluorescein diacetate (DCF-DA) was supplied from Sigma-Aldrich (St. Louis, MO, USA). ICG was supplied from TCI (Tokyo, Japan). PTX was obtained from LC Laboratories (Woburn, MA, USA). A Sonicator 740 (Mettler Electronics Corp., Anaheim, CA, USA) was used for US irradiation. Male CAnN.Cg-Foxn1 nu/Crl mice were provided from ORIENT (Gyeonggi-do, Korea).

### Preparation and characterization of EVs

EVs were fabricated and isolated from HEK-293T human embryonic kidney cells adopting ExoQuick-TC™ (System Biosciences, Palo Alto, USA). The cells were maintained with EV-free FBS (Gibco, Thermo Fisher Scientific, Waltham, MA, USA) for 3 days to produce EVs. Then, the conditioned media underwent 15 min of centrifugation (3000 *g*) to exclude cellular debris. The supernatant was treated with an ExoQuick-TC™ solution and kept overnight, followed by 30 min of centrifugation (1500 *g*). The pellet containing EVs was washed and then resuspended in PBS. The EVs were stored at -80 °C. Nanoparticle tracking analysis (NTA, Nanosight NS300, Malvern, Worcestershire, UK) was used to measure the concentration of the EVs.

### Fabrication and characterization of engineered EVs

Stock solutions of ICG (20 mg/mL) and PTX (20 mg/mL) were prepared in DMSO. The encapsulation of ICG and PTX into EVs was achieved via a simple incubation method. Briefly, 0.2 mg ICG was mixed with 5 × 10^11^ EVs in 500 µL DMSO/PBS [4% (v/v)] and incubated for 2 h at 4 °C in the dark to produce EV(ICG). Unloaded ICG and bulk proteins were removed by using PD MiniTrap G-25 columns (Cytiva, Marlborough, MA, USA). To prepare SBC-EV(ICG), 2.5 µL of an SBC solution in DI water (1 mM) was added into 500 µL of 4% DMSO (v/v) in PBS containing 0.2 mg ICG and 5 × 10^11^ EVs. The mixture was incubated for 2 h in the dark. The purified SBC-EV(ICG) was obtained using PD G-25 columns. To fabricate EVs co-loaded with ICG and PTX [EV(ICG/PTX)], 0.2 mg of ICG and 0.1 mg of PTX were added into 500 µL of PBS [total of 4% (v/v) DMSO] containing 5 × 10^11^ EVs. The mixture was stirred for 4 h at 4 °C, and the EV(ICG/PTX) was purified using PD G-25 columns. To obtain SBC-EV(ICG/PTX), 2.5 µL of SBC solution in DI water (1 mM) was added to a mixture of 5 × 10^11^ EVs, 0.2 mg ICG, and 0.1 mg PTX in 500 µL PBS containing 4% (v/v) DMSO. The mixture solution was kept for 4 h at 4 °C, followed by rapid purification using PD G-25 columns. The final product was stored at 4 °C. To fabricate SBC-EV(ICG/PTX) with different encapsulation ratios of ICG and PTX, the combination of 0.2 mg ICG, 0.2 mg PTX, and 5 µM SBC solutions was mixed with 5 × 10^11^ EVs in 500 µL PBS containing 4% (v/v) DMSO.

The size of the drug-loaded EVs was measured using NTA (Nanosight NS300). The zeta potential of the engineered EVs was measured using a Nano-ZS Zetasizer (Malvern). The encapsulation efficiencies of ICG and PTX were calculated as: (amount of ICG or PTX loaded into EVs)/(initial amount of ICG or PTX) × 100%. To quantify the amount of ICG and PTX loaded into various EVs, liquid chromatography-tandem mass spectrometry (LC-MS/MS) was conducted. Agilent LC 1100 series equipped with a 6490 triple quadrupole mass spectrometer and an electrospray ionization (ESI) source (Agilent Technologies, Santa Clara, CA, USA) were used to obtain LC-MS/MS results of samples. Acetonitrile and 0.1% formic acid in water [40:60 (v/v) for ICG; 60:40 (v/v) for PTX] were used as an eluent. The encapsulation efficiency of SBC in EVs was assessed by the quantification of sodium ions using ICP-OES (iCAP 7000). Blank EVs were adopted as the negative controls.

### Colloidal stability assessment of SBC-EV(ICG/PTX)

To investigate the colloidal stability of EV(ICG/PTX) or SBC-EV(ICG/PTX), they were suspended in PBS comprising 10% EV-depleted FBS and incubated at 37 °C. The size of the EV(ICG/PTX) or SBC-EV(ICG/PTX) was measured at predetermined time intervals using NTA.

### Assessment of aqueous stability of ICG in ICG-loaded EVs

To assess the aqueous stability of various ICG-loaded EVs, the change in ICG fluorescence was monitored for 14 days. Free ICG, EV(ICG), SBC-EV(ICG), and SBC-EV(ICG/PTX) at an equivalent ICG concentration of 10 μg/mL were suspended in PBS and maintained at 4 °C under normal lighting conditions. The fluorescence intensity of ICG from EV-derived samples was measured by UV-Vis spectroscopy (λ_ex_: 780 nm, λ_em_: 835 nm) (Infinite M200 pro).

### Determination of CO_2_ generation from SBC-loaded EVs under acidic conditions

CO_2_ generation from SBC-EV was assessed by using a commercially available Carbon Dioxide Test Kit (Hanna Instruments, Smithfield, RI, USA). The solution of blank EV and SBC-EV samples were mixed with pH 7.4 or pH 6.0 aqueous solutions in small plastic vessels containing phenolphthalein as an acid-base indicator. The relative levels of CO_2_ generation from SBC-loaded EVs and blank EVs at different pH conditions were determined by the amount of NaOH used to neutralize the mixture solutions, in which CO_2_ dissolves and forms carbonic acid.

### *In vitro* drug release profiles of SBC-EV(ICG/PTX)

To investigate whether pH and US influence the release of PTX and ICG from SBC-EV(ICG/PTX), 100 µL of SBC-EV(ICG/PTX) solution [or EV(ICG/PTX)] at pH 5.0, 6.0, 6.6 or 7.4, was added into a Slide-A-Lyzer Mini Dialysis device (MW cutoff = 20 kDa, Thermo Fisher Scientific). The device was inserted into a microcentrifuge tube (1.5 mL) containing PBS (pH 7.4, 0.9 mL). To investigate the US-responsive drug release of SBC-EV(ICG/PTX), it was treated with US (1 MHz, 0.3 W/cm^2^) for 1 min and incubated in the dark at 37 °C. At certain predetermined time intervals (i.e., 4, 8, and 12 h), the solution in the microcentrifuge tube was taken to quantify the amount released PTX (or ICG). The bottom tube was replaced with a new tube containing fresh PBS. The amount of released PTX (or ICG) in each bottom tube was quantified using LC-MS/MS.

To evaluate how much SBC remains in the SBC-EV(ICG/PTX) under *in vivo*-mimicking conditions, the release profiles of SBC from SBC-EV were evaluated using the same dialysis method above. Blank EV and SBC-EV dispersed in 260 mM KCl solution were introduced into a Mini Dialysis device. At predetermined time intervals (i.e., 1, 2, 4, 8, 12, 24, and 48 h), the amount of released SBC was quantified using ICP-OES (iCAP 7000, Thermo Fisher Scientific). Blank EV was used as a negative control.

### Cellular internalization analysis of free ICG and ICG-loaded EVs

MCF-7 cells were inoculated into 24-well plates (5 × 10^4^ cells per well) 24 h before treatment. Free ICG, EV(ICG), and SBC-EV(ICG) at an equivalent ICG concentration were added to the cells. After 4 h of treatment, the fluorescence of ICG in the cells was measured using a flow cytometer (CytoFLEX), as described in the previous study 70.

To quantify cellular uptake of EV samples by MCF-7 cells, FITC was used to label EVs. Subsequently, 0.4 µg of FITC was added into 500 µL PBS-containing 1 × 10^12^ EVs and kept for 2 h at 4 °C in the dark. The final product was purified using PD G-25 columns. FITC-EV(ICG) and FITC-SBC-EV(ICG) were prepared in the same manner used for the preparation of EV(ICG) and SBC-EV(ICG). MCF-7 cells were treated with various samples and incubated for 4 h. The cellular uptake of FITC-labeled EV samples was determined by quantifying the fluorescence signal from FITC, as described in the previous study 70.

### Live cell imaging using confocal laser scanning microscopy (CLSM)

To observe intracellular localization of EV(ICG) and SBC-EV(ICG) by confocal laser scanning microscopy, RB-labeled EVs were prepared. 0.4 μg RB isothiocyanate (Sigma Aldrich) was mixed with 1 × 10^12^ EVs in 500 µL PBS. The mixture was stirred in the dark for 2 h at 4 ºC. The crude product was purified from unreacted RB using PD G-25 columns. RB-labeled EV(ICG) and RB-labeled SBC-EV(ICG) were fabricated and purified according to the method mentioned above for the preparation EV(ICG) and SBC-EV(ICG). RB-labeled EVs were used instead of EVs for the preparation of RB-labeled EV samples.

For CLSM, MCF-7 cells were seeded into a glass-bottom dish (35×10 mm) (Eppendorf, Hamburg, Germany) at a concentration of 2 ×10^3^ cells per well and incubated for 24 h. The cells were stained with 1 μM LysoTracker^TM^ Green DND-26 (Thermo Fisher Scientific) and 1 μg/mL Hoechst 33342 (Thermo Fisher Scientific) at 37 ºC for 10 min. Then, the glass bottom dish was placed in live cell chamber systems (5% CO_2_ at 37 ºC) connected to a confocal laser scanning microscope (Al Plus, Nikon, Japan). The cells were treated with RB-labeled EV(ICG) and RB-labeled SBC-EV(ICG) at an equivalent ICG concentration and then immediately observed using a 60× oil immersion objective. Live cell images were acquired with z section of 0.1 mm thickness over 1 h period. The fluorescent images were analyzed using Nikon NIS-E image analysis software. The Mander's overlap coefficients were obtained to quantify the degree of colocalization between fluorescence signals.

### ROS generation using ICG-loaded EVs upon US treatment

The US-triggered ROS generation using free ICG and ICG-loaded EVs in a cell-free system was determined by 2',7'-dichlorofluorescein (DCF). DCF was prepared by reacting DCF-DA with KOH (0.1 mM). ICG-loaded EVs and free ICG and solutions were added to a 10 µM DCF solution. Then, the mixture solutions in microcentrifuge tubes were exposed to 1 min of US (1 MHz, 0.3 W/cm^2^). After further incubation in the dark for 1 h, the green fluorescence intensity of the sample solution was measured (λ_ex_: 495 nm, λ_em_: 529 nm). Samples that did not receive US treatment were used as controls.

The intracellular ROS production in the cells incubated with free ICG and ICG-loaded EVs was quantified before and after US irradiation. MCF-7 cells were prepared in 24-well plates (5 × 10^4^ cells/well) 24 h before treatment. Then, the cells were washed with PBS and treated with a 10 µM DCF-DA solution for 30 min. After the cells were rinsed with PBS, they received free ICG (28 µg/mL), free PTX, EV(ICG) (28 µg/mL ICG), SBC-EV(ICG) (28 µg/mL ICG), and SBC-EV(ICG/PTX) (28 µg/mL ICG, 10 µg/mL PTX). After 4 h of treatment, the cells were separated into two groups (control group and US-treated group). 1 MHz US was irradiated to the cells for 1 min (0.3 W/cm^2^). After 30 min of incubation, intracellular ROS levels in the cell were quantified in the same manner as previously reported 70.

To observe the US-triggered ROS generation in MCF-7 cells, the cells were inoculated into 24-well plates and stained with 10 µM DCF-DA. The DCF-DA-stained cells were treated with free ICG and SBC-EV(ICG/PTX) (28 µg/mL ICG, 10 µg/mL PTX) for 4 h. Then, the treatment solution was replaced with PBS. For the US-treated samples, the bottom of the well plate with a layer of ultrasound gel was irradiated with 1 MHz US (0.3 W/cm^2^) for 1 min. After 30 min of incubation, the ROS generation from the cells was observed using a fluorescence microscope (Nikon Eclipse Ti-S, Nikon, Tokyo, Japan).

### *In vitro* sonotoxicity evaluation

MCF-7 cells were cultured in 96-well plates (1 × 10^4^ cells/well). Free ICG (28 μg/mL ICG), EV(ICG) (28 μg/mL of ICG), SBC-EV(ICG) (28 μg/mL ICG), EV(ICG/PTX) (28 μg/mL ICG and 10 μg/mL PTX, 5.59 × 10^10^ EVs/mL), and SBC-EV(ICG/PTX) (28 μg/mL ICG and 10 μg/mL PTX, 5.88 × 10^10^ EVs/mL) were added to the cells. After 4 h of incubation, the cells were incubated with a fresh culture medium [minimum essential medium/Earle's balanced salt solutions (MEM/EBSS)/10% FBS], followed by irradiation with 1 MHz US at 0.3 W/cm^2^ for 1 min. The cell viability was analyzed by a conventional MTT assay after 24 h of incubation 70.

### *In vitro* apoptosis analysis and live/dead cell staining

MCF-7 cells were seeded in 12-well plates (3 × 10^5^ cells/well) 24 h before treatment. Then, the cells were incubated with free ICG (28 μg/mL ICG) and SBC-EV(ICG/PTX) (28 μg/mL ICG, 5.88 × 10^10^ EVs/mL) for 4 h. After the sample treatment, the cells were rinsed with PBS and suspended in culture media. Then, the suspension was divided in half, and one-half received US treatment at 0.3 W/cm^2^ for 1 min. The other half was adopted as the control. The cells were seeded back into 12-well plates, followed by 8 h of incubation. Then, the cells were trypsinized and resuspended in binding buffer for further staining with Annexin V-FITC and PI (BD Biosciences, Heidelberg, Germany). Annexin V-positive and PI-negative cells were identified as early apoptotic cells. Both Annexin V- and PI-positive cells were identified as late apoptotic cells. Viable cells were negative for both strains.

For live and dead cell staining, MCF-7 cells were separately prepared in different 12-well plates (3 × 10^5^ cells/well) for US-treated and untreated groups. The cells that received free ICG and SBC-EV(ICG/PTX) (28 μg/mL ICG, 5.88 × 10^10^ EVs/mL) were incubated for 4 h. Then, the solution in the well was replaced with a fresh culture medium, followed by US treatment for 1 min (0.3 W/cm^2^). After 8 h of incubation, the cells were rinsed with PBS and stained with 1 μM calcein-AM (Thermo Fisher Scientific) and 2 μM ethidium homodimer-1 (Thermo Fisher Scientific) according to the manufacturer's instructions. Fluorescent images of the cells were acquired using a fluorescence microscope (Nikon Eclipse Ti-S).

### Real-Time PCR (qRT-PCR)

To assess the effect of PTX on ROS generation-related mRNA expression, real-time polymerase chain reaction (qRT-PCR) was performed using the StepOnePlus Real-Time PCR System (Applied Biosystems, USA). MCF-7 cells were incubated with PTX for 2 h, followed by the activation of NADPH oxidase. RNA was isolated using Tris-RNA reagent (Favorgen, Taiwan), according to the manufacturer's protocol. cDNA was synthesized using ReverTra Ace qPCR RT Master Mix (Toyobo, Japan), and RT-PCR was performed using the THUNDERBIRD SYBR qPCR mix (Toyobo, Japan). NADPH oxidase 1 (NOX1) and β-actin as an internal control were analyzed with the primers listed in Table S3.

### MCF-7 tumor-bearing mouse models for *in vivo* studies

All animal experiments were performed under a protocol approved by the Institutional Animal Care and Use Committee at Gachon University, Republic of Korea (LCDI-2017-0131). We subcutaneously injected 5-week-old BALB/c nude mice with MCF-7 cells (1 × 10^6^ cells in 100 µL of PBS) into the right back region. The mice were randomized into each sample group (*n* = 4) when the tumor volume reached approximately 100 mm^3^.

### *In vivo* biodistribution study using *in vivo* imaging systems (IVIS)

For *in vivo* real-time biodistribution imaging of SBC-EV(ICG/PTX) and free ICG, MCF-7 tumor-xenograft mice were intravenously injected with 100 μL of free ICG and SBC-EV(ICG/PTX) (1.0 mg ICG/kg and 0.4 mg PTX/kg). The* in vivo* biodistribution imaging was carried out using an IVIS system (Ami HT imaging system, Spectral Instruments Imaging, Tucson, AZ, USA) by measuring the ICG fluorescence intensity of whole body with a long wave emission filter (745-830 nm) at predetermined time intervals within 24 h. The mice were euthanized at 4 h or 24 h-post injection to obtain *ex vivo* fluorescent images of major organs and tumors of the mice.

### *In vitro* and *in vivo* PA imaging

*In vitro* and* in vivo* PA imaging studies were conducted using previously reported methods 71. In addition, a Q-switched pump laser (Surelite III-10, Continuum, USA; wavelength: 532 nm or 1064 nm) and an optical parametric oscillator laser (Surelite OPO PLUS, Continuum, USA; wavelength: 680-930 nm; pulse repetition frequency: 10 Hz; pulse width: 5 ns) were used, and the two lasers were synthesized. The wavelength was set to 780 nm for PA imaging of the absorption peak of ICG 72. The transmitted light intensity was approximately 5 mJ/cm^2^, which is lower than the safety limit of the American National Standards Institute (ANSI) at 780 nm. The PA signal generated by the laser was detected using a 5 MHz US transducer (V308, Olympus NDT, Waltham, MA, USA). For *in vitro* experiments, free ICG, EV(ICG/PTX), and SBC-EV(ICG/PTX) at equivalent concentrations of ICG were injected into silicone tubes (Dow Corning) from 15.625 to 1000 μg/mL, and the PA signals were then measured at 780 nm.

MCF-7 tumor-xenograft mice received 150 μL of free ICG (0.8 mg/mL ICG) and EV(ICG/PTX) (0.8 mg/mL ICG and 0.3 mg/mL PTX) via i.v. injection (*n* = 3). To facilitate the PA imaging process, fine hairs around the tumor were removed using a depilatory cream, and the mice were placed under a water tank piled up with plastic wrap for acoustic impedance matching. Acoustic-resolution photoacoustic microscopy (AR-PAM) was utilized for imaging. The scanning area was 60 × 40 mm^2^, and the step sizes were 0.4 mm in the x and y directions, respectively. It took approximately 45 min to obtain a single whole-body PA image. Whole-body PA images of mice were obtained at 0 h and 4 h post-injection. The top-view MAP images were obtained by calculating the data obtained, and depth-coded MAP images were acquired over time.

AR-PAM was used for *in vivo* PA imaging of tumors. The water tank was wrapped to minimize the impedance gap. MCF-7 tumor-bearing mice received 150 μL of SBC-EV(ICG/PTX) (0.8 mg/mL ICG) via i.v. injection. Fine hairs around the tumor were removed in advance using a depilatory cream, and a thermal mat was used to maintain the body temperature. The scanning area was set to 15 × 15 mm^2^, and the step sizes were 0.4 mm in the *x* and *y* directions. Tumor PA images of mice were obtained at 1 h and 4 h post-injection. The top-view MAP images were obtained.

### *In vivo* antitumor efficacy of SBC-EV(ICG/PTX) with US treatment

MCF-7 tumor-bearing mice received one of the following regimens through the tail vein injection: (i) PBS; (ii) free ICG and free PTX (1.0 mg ICG/kg and 0.4 mg PTX/kg); (iii) EV(ICG/PTX) (1.0 mg ICG/kg and 0.4 mg PTX/kg); (iv) SBC-EV(ICG/PTX) (1.0 mg ICG/kg and 0.4 mg PTX/kg). After 4 h post-i.v. injection, 3 min of US (1 MHz, 0.5 W/cm^2^) was irradiated to the tumor site of mice. The tumor volumes [(tumor length) × (tumor width)^2^/2] were measured every other day. In addition, to evaluate the safety of the engineered EVs, each mouse was weighed every other day. Two weeks after the sample treatment and US irradiation, blood was collected from the saphenous vein into a potassium ethylenediamine tetra-acetic acid collection tube for blood analysis. Then, the mice were sacrificed, and tumor xenografts and major organs including the heart, liver, lungs, kidneys, and spleen were isolated and fixed with 4% paraformaldehyde. Conventional H&E staining was conducted to stain tissue sections.

### Statistical analysis

All the data were acquired in triplicate unless specified otherwise. Data were indicated as mean ± standard deviation (SD) values. Statistical significance between sample groups was determined by one-way ANOVA and represented: *p < 0.05; **p < 0.01.

## Figures and Tables

**Table 1:** Drug encapsulation efficiency of ICG in SBC-EV(ICG/PTX) with different DMSO composition ratios (v/v) in PBS for the preparation

DMSO composition ratio (v/v)	0%	1%	2%	3%	4%
ICG encapsulation efficiency (%)	23.4±1.8	36.8±0.8	38.2±0.2	41.7±0.8	42.9±3.5
PTX encapsulation efficiency (%)	N/A	5.7±3.6	19.6±0.5	32.4±1.3	39.9±1.2

**Table 2:** Size and surface charges of various EV-derived samples

Samples	Size (nm)	Zeta potential (mV)
Blank EV	117.1±2.0	-19.6±1.2
SBC-EV	121.1±3.2	-20.2±0.5
EV(ICG)	129.6±3.1	-20.1±0.3
SBC-EV(ICG)	132.7±6.8	-23.6±2.5
EV(ICG/PTX)	149.9±5.2	-22.7±1.2
SBC-EV(ICG/PTX)	150.8±4.2	-26.4±2.4

## Abbreviations

ROS: reactive oxygen species
PDT: photodynamic therapy
SDT: sonodynamic therapy
US: ultrasound
EPR: enhanced permeability and retention
PA: photoacoustic
NIR: near-infrared
ICG: indocyanine green
PTX: paclitaxel
SBC: sodium bicarbonate
EV: extracellular vesicle
DCF-DA: 2',7'-dichlorofluorescein diacetate
RB: rhodamine B
NADPH: nicotinamide adenine dinucleotide phosphate
ICP-OES: inductively coupled plasma optical emission spectrometry
NTA: nanoparticle tracking analysis
MAP: maximum amplitude projection
AR-PAM: acoustic-resolution photoacoustic microscopy
