# Supplementary material for: Engineered extracellular vesicle-based sonotheranostics for dual stimuli-sensitive drug release and photoacoustic imaging-guided chemo-sonodynamic cancer therapy
Source: Theranostics. 2022 Jan 1;12(3):1247–66. doi: 10.7150/thno.65516 (PMC8771566; doi:10.7150/thno.65516)
Supplement: Supplementary file 1 — Supplementary figures and tables. [file thnov12p1247s1.pdf]

# Supporting Information

# Engineered extracellular vesicle-based sonotheranostics for dual stimuli-sensitive drug release and photoacoustic imaging-guided chemo-sonodynamic cancer therapy

Thuy Giang Nguyen Cao[1, †], Ji Hee Kang[2,†], Wangyu Kim[3,†], Junha Lim[4,†], Su Jin Kang[1], Jae Young You[1], Quan Truong Hoang[1], Won Jong Kim[4], Won Jong Rhee[1,5]*, Chulhong Kim[3,]*,Young Tag Ko[2,]*, Min Suk Shim[1,]*

[1]Division of Bioengineering, Incheon National University, Incheon 22012, Republic of Korea

[2]College of Pharmacy, Gachon University, Incheon 21936, Republic of Korea

[3]Department of Electrical Engineering, Convergence IT Engineering, and Mechanical Engineering, Medical Device Innovation Center, Pohang University of Science and Technology (POSTECH), Pohang 37673, Republic of Korea

[4]Department of Chemistry, Pohang University of Science and Technology (POSTECH), Pohang 37673, Republic of Korea

[5]Research Center for Bio Materials & Process Development, Incheon National University, 119 Academy-ro, Yeonsu-gu, Incheon 22012, Republic of Korea

[†] These authors contributed equally to this work.

*Corresponding authors:

Dr. Won Jong Rhee

Tel: +82-32-835-8299; E-mail address: wjrhee@inu.ac.kr

Dr. Chulhong Kim

Tel: +82-54-279-8805; E-mail address: chulhong@postech.ac.kr

Dr. Young Tag Ko

Tel: +82-32-820-4923; E-mail address: youngtakko@gachon.ac.kr

Dr. Min Suk Shim

Tel: +82-32-835-8268; E-mail address: msshim@inu.ac.kr

**Table S1.** $IC_{50}$ values of ICG and PTX in various SBC-EV(ICG/PTX) samples against MCF-7 cells before and after US irradiation.

| Samples | ICG (μg/mL) | PTX (μg/mL) |
|---|---|---|
| Free ICG | 212.56±9.00 | - |
| Free ICG + US | 197.08±10.54 | - |
| Free PTX | - | 15.95±0.28 |
| Free PTX + US | - | 15.16±0.09 |
| SBC-EV(ICG/PTX) (28:2) | 185.58±3.82 | 13.26±0.27 |
| SBC-EV(ICG/PTX) (28:2) + US | 174.49±3.74 | 12.46±0.73 |
| SBC-EV(ICG/PTX) (28:4) | 91.71±0.54 | 12.01±0.25 |
| SBC-EV(ICG/PTX) (28:4) + US | 75.41±2.23 | 10.78±0.32 |
| SBC-EV(ICG/PTX) (28:10) | 27.04±0.16 | 9.66±0.06 |
| SBC-EV(ICG/PTX) (28:10) + US | 17.17±0.02 | 6.13±0.01 |
| SBC-EV(ICG/PTX) (28:13) | 14.22±0.39 | 6.49±0.07 |
| SBC-EV(ICG/PTX) (28:13) + US | 12.99±0.21 | 5.95±0.08 |
| SBC-EV(ICG/PTX) (28:18) | 9.77±0.07 | 6.27±0.05 |
| SBC-EV(ICG/PTX) (28:18) + US | 9.26±0.27 | 5.81±0.09 |
| SBC-EV(ICG/PTX) (28:22) | 7.95±0.11 | 6.24±0.09 |
| SBC-EV(ICG/PTX) (28:22) + US | 7.08±0.03 | 5.56±0.02 |

**Table S2.** The area under the plasma concentration-time curve (AUC) of ICG from tumor-bearing mice ($n$ = 4) after intravenous injection of free ICG and EV(ICG/PTX) (%ID: percentage of an injected dose).

| Samples | AUC (%ID·h/mL) |
|---|---|
| Free ICG | 0.7±0.3 |
| EV(ICG/PTX) | 4.0±0.5 |

**Table S3.** qRT-PCR primer sequences.

| **Samples** | **qRT-PCR primers** |
|---|---|
| NOX1 | Forward: GGTTTTACCGCTCCCAGCAGAA<br>Reverse: CTTCCATGCTGAAGCCACGCTT |
| β-actin | Forward: ATGAAGTGTGACGTTGACATCCG<br>Reverse: GCTTGCTGATCCACATCT |

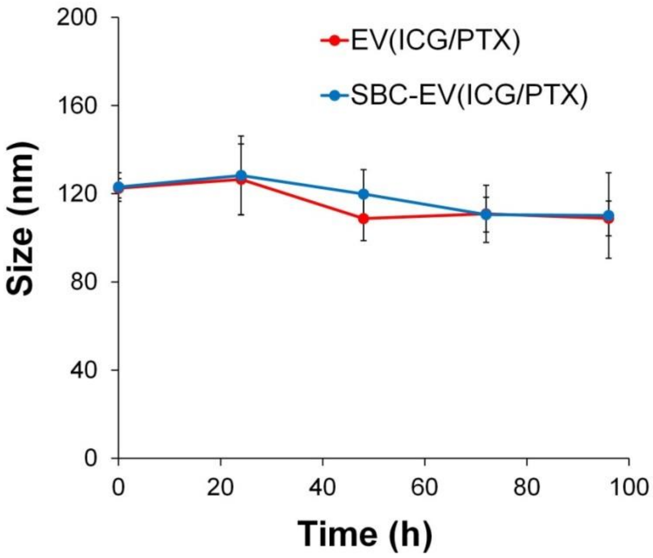


**Figure S1.** Size changes of EV(ICG/PTX) and SBC-EV(ICG/PTX) incubated in 10% FBS-containing PBS for 96 h.

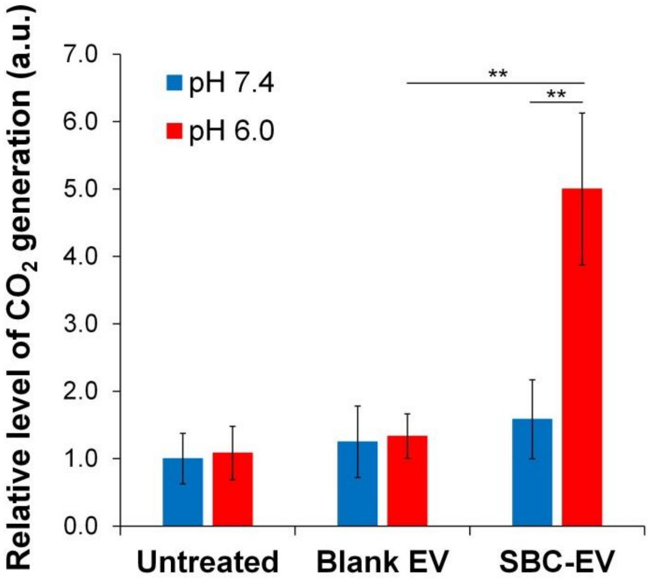


**Figure S2**. Relative levels of $CO_2$ generation by blank EV and SBC-EV under different pH conditions, determined by an acid-base titration method (***p* < 0.01; *n* = 3).

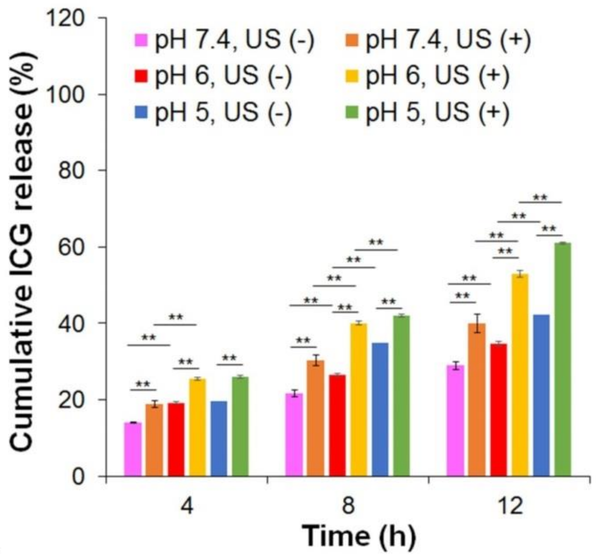


**Figure S3.** ICG release profiles from SBC-EV(ICG/PTX) at different pH and US (1 min of irradiation) conditions (**$p$ < 0.01; $n$ = 3).

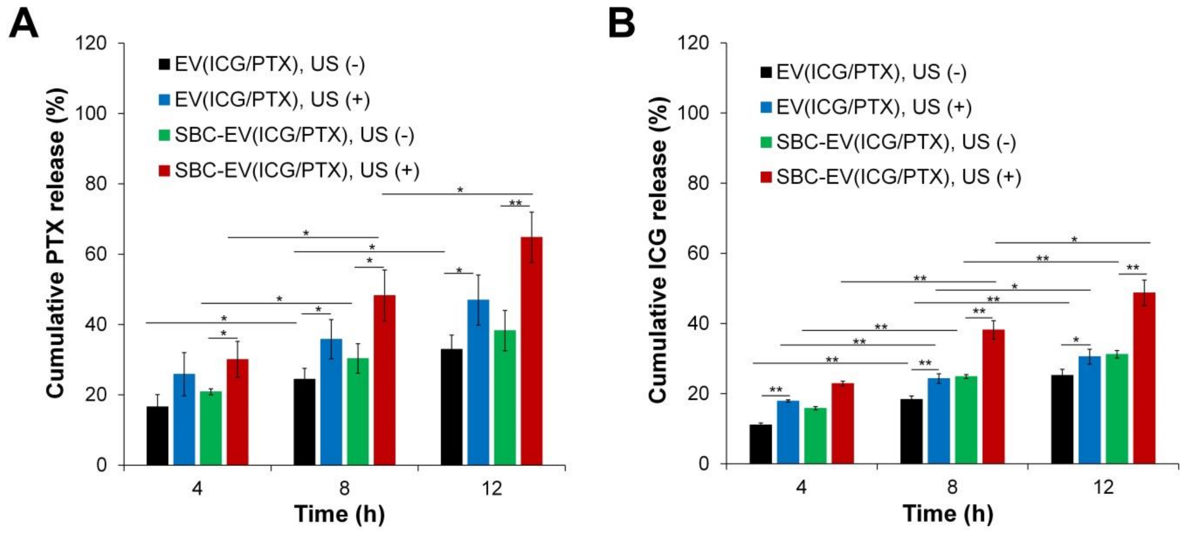


**Figure S4.** Release profiles of PTX and ICG from SBC-EV(ICG/PTX) at pH 6.6 and US (1 min of irradiation) conditions (*$p$ < 0.05, **$p$ < 0.01; $n$ = 3).

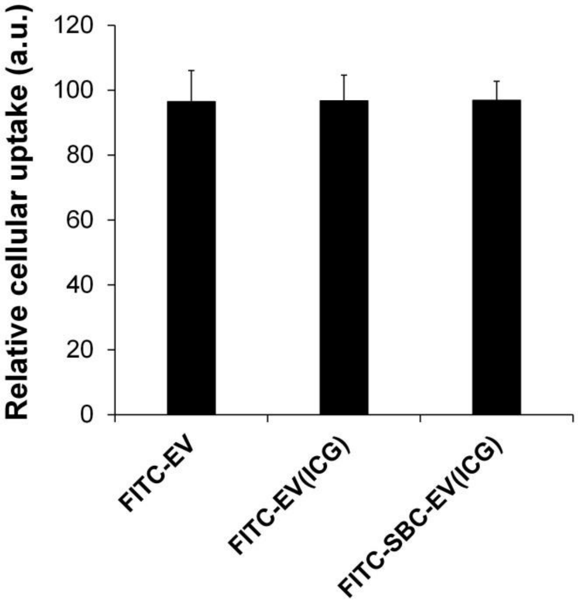


**Figure S5.** Relative cellular uptake of various FITC-labeled EVs by MCF-7 cells after 4 h of incubation.

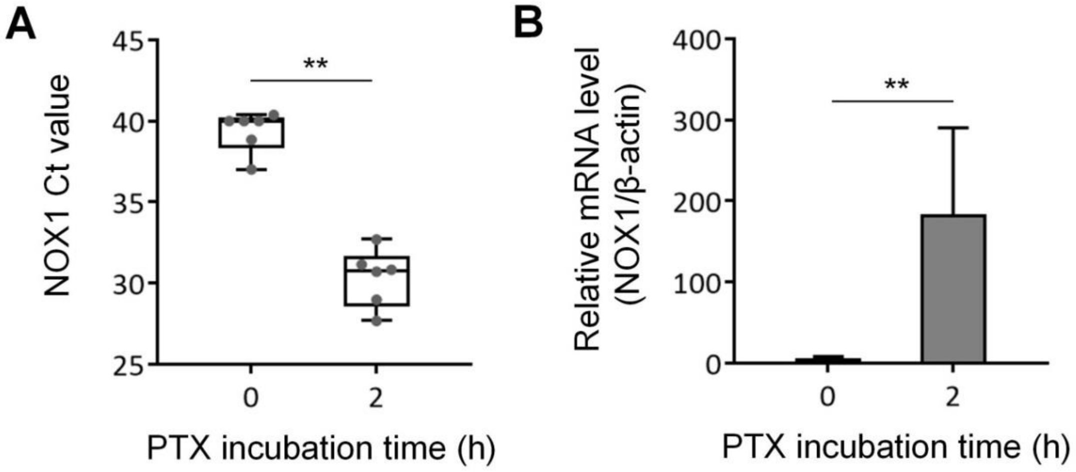


**Figure S6.** The effect of PTX on NOX1 expression in MCF-7 cells. Cycle threshold (Ct) values of NOX1 mRNA (A) and relative NOX1 mRNA expression level (B) in MCF-7 cells after treatment with free PTX were analyzed. $^{**}p < 0.01$ ($n = 6$).

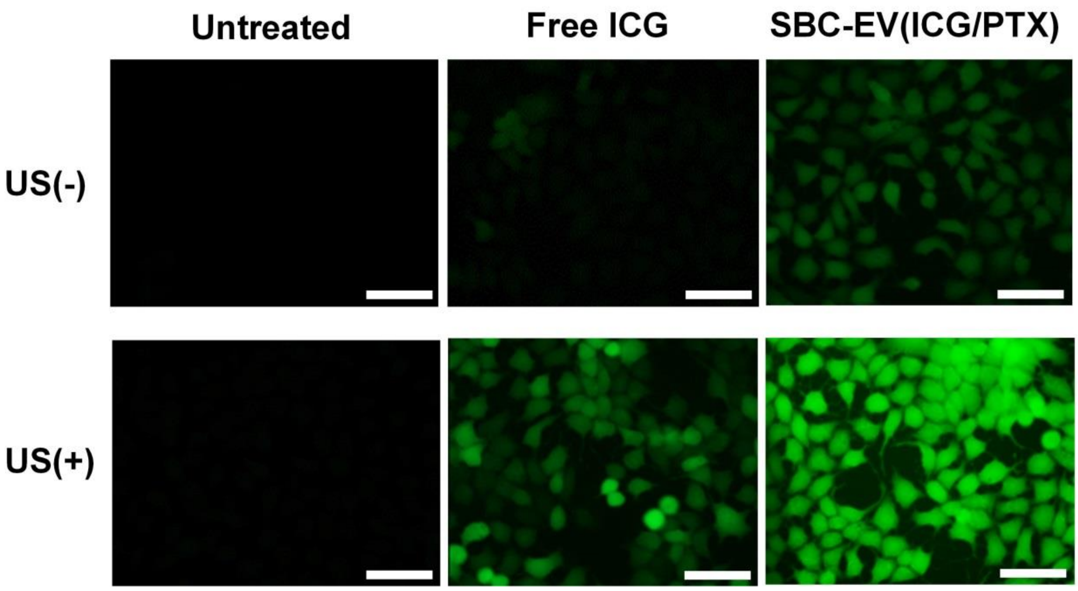


**Figure S7.** Fluorescence images of MCF-7 cells generating intracellular ROS after treatment with free ICG and SBC-EV(ICG/PTX) under US exposure (1 MHz, 0.3 W/$cm^2$, 1 min). Scale bars indicate 25 μm.

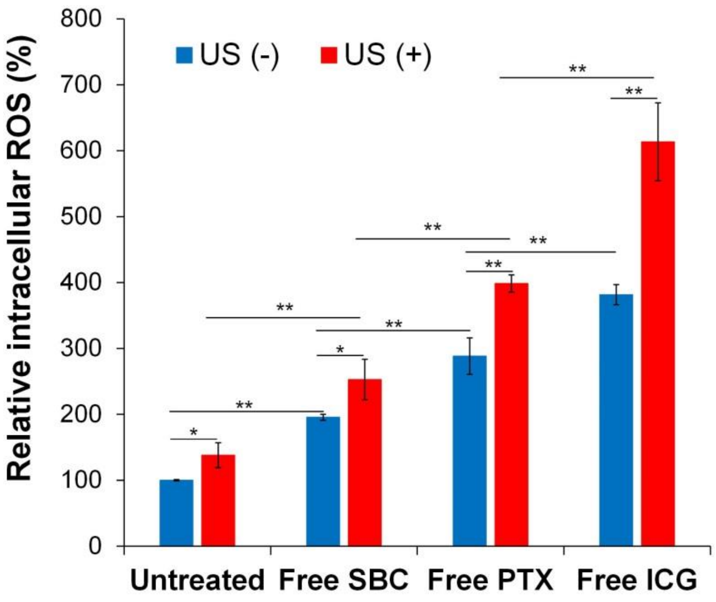


**Figure S8.** Relative intracellular ROS levels of MCF-7 cells treated with free SBC (4 nM), free PTX (10 μg/mL), and free ICG (28 μg/mL) before and after US irradiation (1 MHz, 0.3 W/$cm^2$, 1 min) (*$p < 0.05$, **$p < 0.01$, $n = 3$).

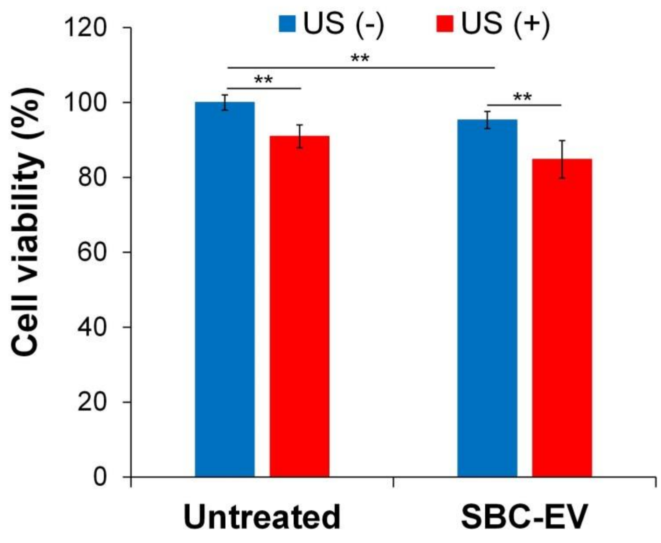


**Figure S9.** Viabilities of MCF-7 cells treated with SBC-EV at 4 nM of SBC concentration before and after US irradiatiion (0.3 W/cm$^2$, 1 min) (**$p$ < 0.01, $n$ = 3).

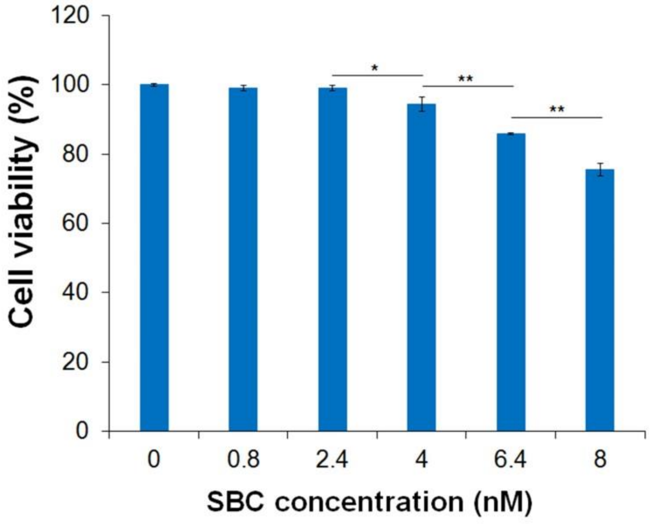


**Figure S10.** Viabilities of MCF-7 cells treated with SBC-EV at different concentrations of SBC (*$p$ < 0.05, **$p$ < 0.01, $n$ = 3).

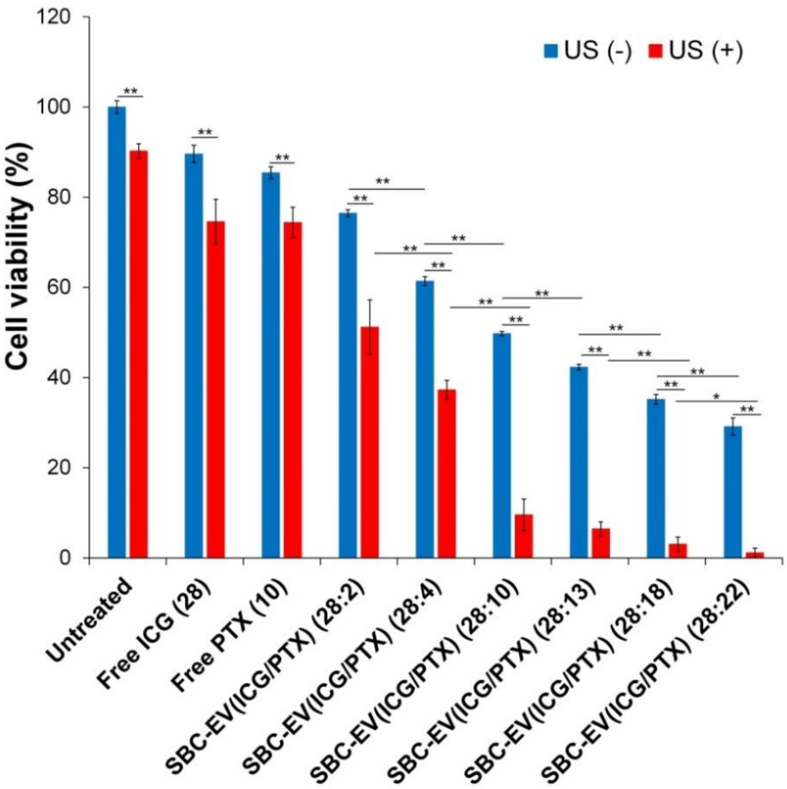


**Figure S11.** Viabilities of MCF-7 cells treated with various SBC-EV(ICG/PTX) at different concentrations of ICG and PTX before and after US irradiation (0.3 W/cm$^2$, 1 min). The number indicates concentrations (μg/mL) of ICG and PTX in the SBC-EV(ICG/PTX) (*$p$ < 0.05, **$p$ < 0.01, $n$ = 3).

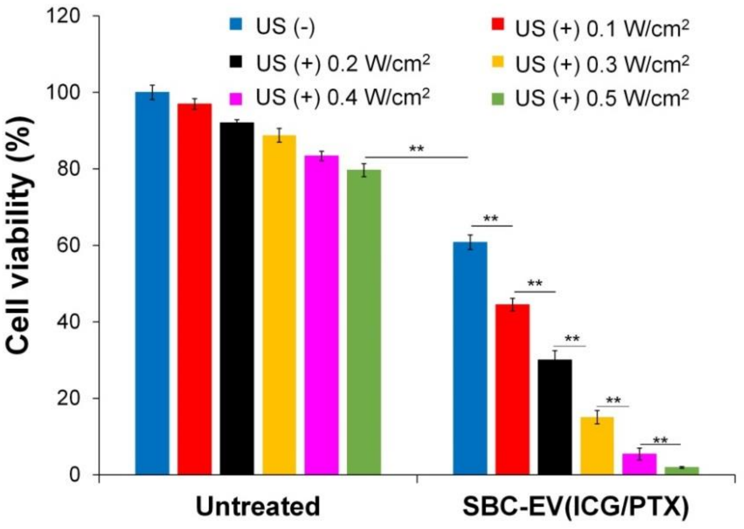


**Figure S12.** Viabilities of MCF-7 cells treated with SBC-EV(ICG/PTX) after irraidation with various intensities of US (**$p$ < 0.01, $n$ = 3).

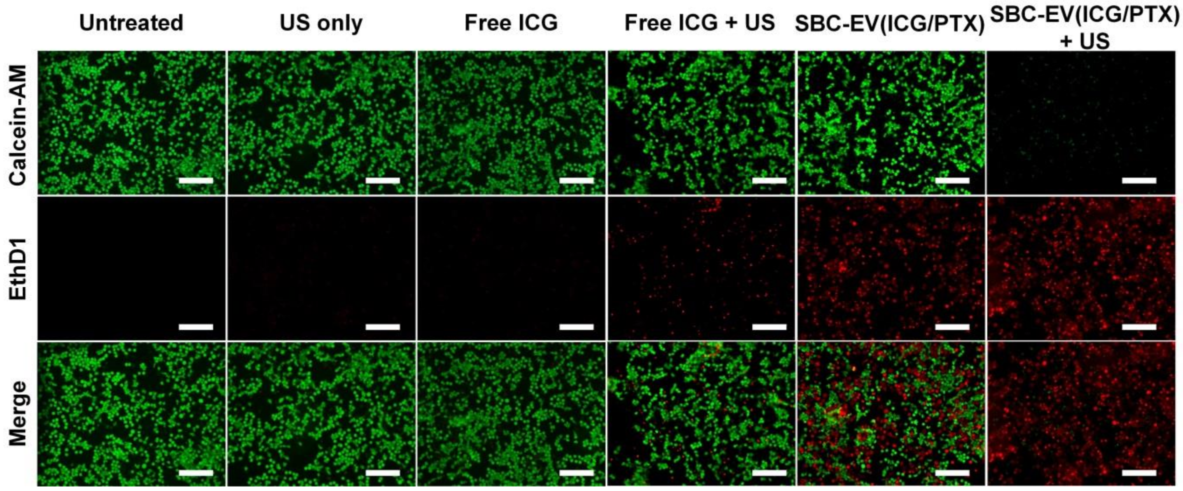


**Figure S13.** Live (green color) and dead (red color) cell staining of MCF-7 cells treated with free ICG and SBC-EV(ICG/PTX) before and after after US irradiation (0.3 W/cm$^2$, 1 min). Scale bars indicate 200 μm.

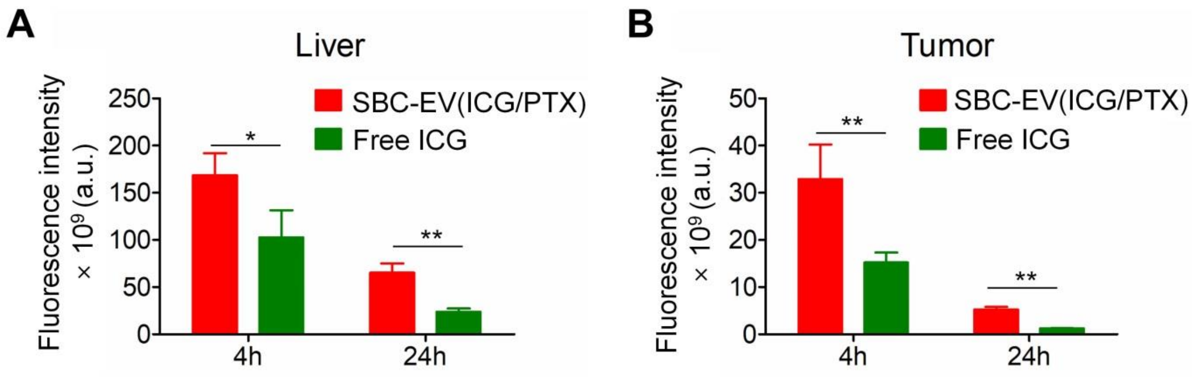


**Figure S14.** Quantitation of ICG from *ex vivo* imaging of liver (A) and tumor (B) at 4 h and 24 h-post i.v. administration of free ICG and SBC-EV(ICG/PTX) (*$p$ < 0.05, **$p$ < 0.01).

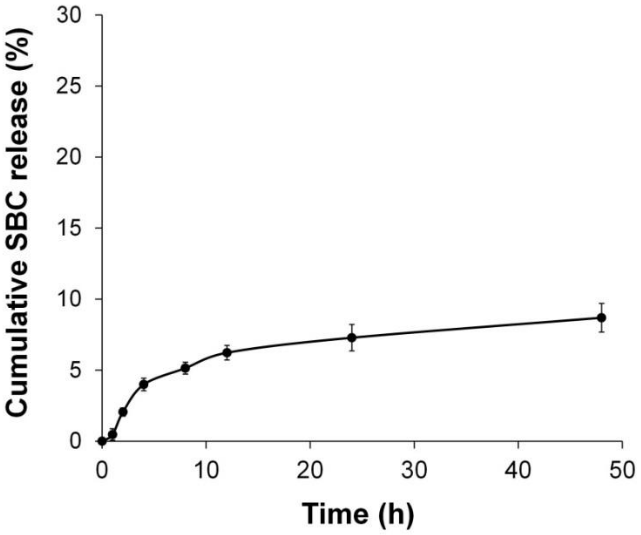


**Figure S15.** Release profiles of SBC from SBC-EV(ICG/PTX) at high salt concentrations (i.e., 260 mM KCl aqueous solution) mimicking in vivo conditions.

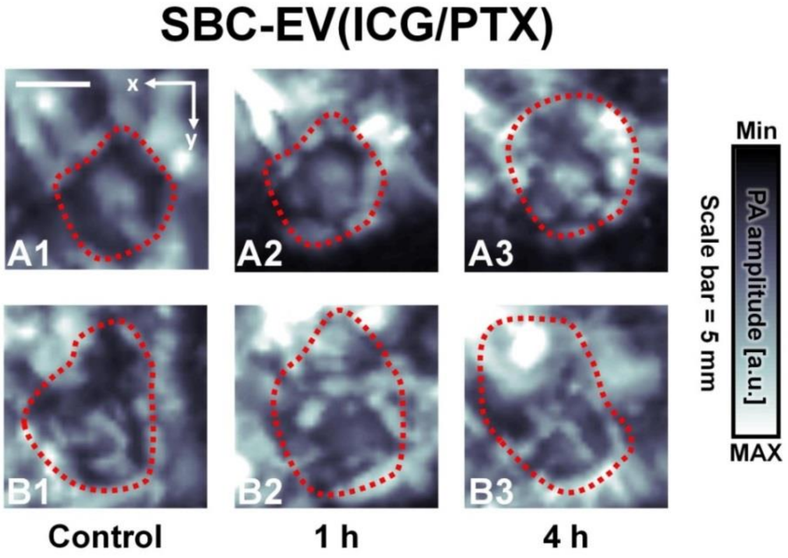


**Figure S16.** *In vivo* PA MAP images of tumors of MCF-7-bearing mice (A and B) at different time intervals after i.v. injection of SBC-EV(ICG/PTX). (MAP: maximum amplitude projection). Scale bar indicates 5 mm. The red circles in the images indicate tumor regions.

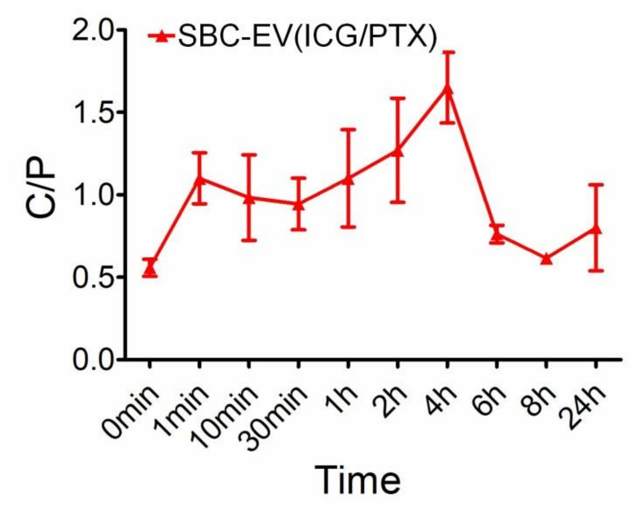


**Figure S17.** The time course ratios of the fluorescence intensity in the tumor core area to the peritumoral tissue area (C/P) in the SBC-EV(ICG/PTX)-treated mice as a function of time, determined by using IVIS.

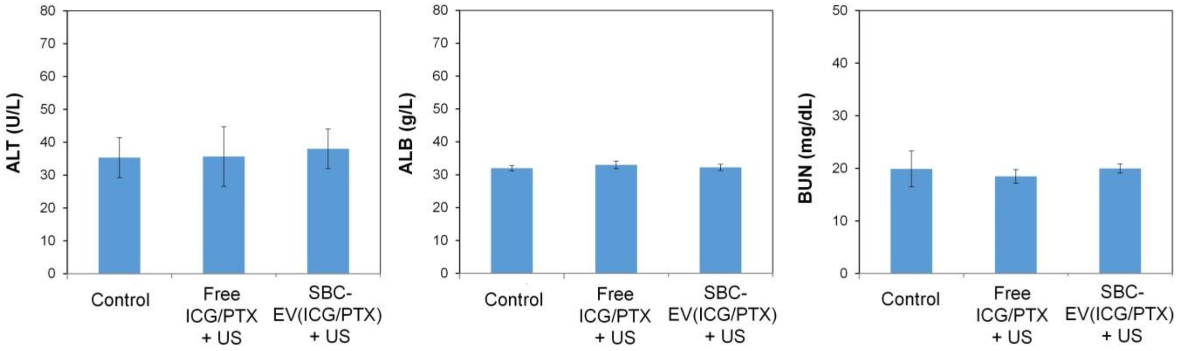


**Figure S18.** Serum biochemistry results obtained from mice injected with free ICG/PTX and SBC-EV(ICG/PTX) at day 14 after i.v. injection. The tumors of mice were irradiated with 3 min of US (1 MHz, 0.5 W/cm$^2$) at 4 h post-injection. These results show mean and standard deviations of alanine aminotransferase (ALT), albumin (ALB), and blood urea nitrogen (BUN).
